# Amino Acid and Phospholipid Metabolism as an Indicator of Inflammation and Subtle Cardiomyopathy in Patients with Marfan Syndrome

**DOI:** 10.3390/metabo11120805

**Published:** 2021-11-27

**Authors:** Lisa Bartenbach, Thomas Karall, Jakob Koch, Markus Andreas Keller, Herbert Oberacher, Sabine Scholl-Bürgi, Daniela Karall, Gregor Oemer, Daniela Baumgartner, Katharina Meinel, Safwat Aly, Irena Odri-Komazec, Ralf Geiger, Miriam Michel

**Affiliations:** 1 Department of Child and Adolescent Health, Division of Pediatrics III—Cardiology, Pulmonology, Allergology and Cystic Fibrosis, Medical University of Innsbruck, 6020 Innsbruck, Austria; lisa.bartenbach@gmx.at (L.B.); thomas.karall@tirol-kliniken.at (T.K.); irena.odri-komazec@tirol-kliniken.at (I.O.-K.); ralf.geiger@tirol-kliniken.at (R.G.); 2 Institute of Human Genetics, Medical University of Innsbruck, 6020 Innsbruck, Austria; jakob.koch@i-med.ac.at (J.K.); markus.keller@i-med.ac.at (M.A.K.); gregor.oemer@gmail.com (G.O.); 3 Institute of Legal Medicine and Core Facility Metabolomics, Medical University of Innsbruck, 6020 Innsbruck, Austria; herbert.oberacher@i-med.ac.at; 4 Department of Child and Adolescent Health, Division of Pediatrics I—Inherited Metabolic Disorders, Medical University of Innsbruck, 6020 Innsbruck, Austria; sabine.scholl-buergi@tirol-kliniken.at (S.S.-B.); daniela.karall@i-med.ac.at (D.K.); 5 Department of Pediatrics, Clinical Division for Pediatric Cardiology, Medical University of Graz, 8010 Graz, Austria; daniela.baumgartner@medunigraz.at (D.B.); katharina.meinel@medunigraz.at (K.M.); 6 The Hospital for Sick Children, Labatt Family Heart Centre, Division for Pediatric Cardiology, Toronto, ON M5G 1X8, Canada; safwat.aly@sickkids.ca

**Keywords:** cardiomyopathy, Marfan syndrome, metabolomics, taurine

## Abstract

Patients with Marfan syndrome (MFS) have an increased risk of aortic aneurysm formation, dissection and development of a subtle cardiomyopathy. We analyzed amino acid and lipid metabolic pathways in MFS patients, seeking biomarker patterns as potential monitoring tools of cardiovascular risk with deterioration of myocardial function. We assessed myocardial function in 24 adult MFS patients and compared traditional laboratory values and mass spectrometry-based amino acid, phospholipid and acylcarnitine metabolomes in patients with those in healthy controls. Analytes for which values differed between patients and controls were subjected to regression analysis. A high proportion of patients had signs of impaired diastolic function and elevated serum levels of NT-proBNP. Patients had lower serum levels of taurine, histidine and PCaeC42:3 than controls. The evidence of diastolic dysfunction, aortic root dimensions and history of aortic root surgery correlated with NT-proBNP and taurine levels. Alterations in serum levels of metabolism derived analytes link MFS pathophysiology with inflammation, oxidative stress and incipient cardiomyopathy.

## 1. Introduction

Marfan syndrome (MFS) is an autosomal-dominant systemic disorder of connective tissue. Its phenotypic continuum is broad, ranging from mild features of MFS in one or a few systems to severe and rapidly progressive neonatal multiorgan disease. Its incidence is 1:5000 newborns worldwide, with 75% born to an affected parent and 25% resulting from de novo mutation [1,2,3].

MFS is caused by mutations in *FBN1*, encoding the glycoprotein fibrillin-1. Assemblages of fibrillin-1 form microfibrils in the extracellular matrix, providing elasticity and structural integrity. By storing or releasing the critical agent transforming growth factor β (TGF-β), these microfibrils regulate its bioavailability; TGF-β usually is secreted in an inactivated (latent) form that requires proteolysis to permit induction of collagen synthesis and, in counterbalance, of matrix metalloproteinase (MMP) activity to degrade extracellular matrix proteins [3]. Mutated fibrillin-1 forms microfibrils poorly, with failed sequestration of latent TGF-β. Excessive activation of TGF-β signaling cascades follows. Thus, mutations in *FBN1* and consequent deficiency in fibrillin-1, as in MFS, result not only in structural weakness of connective tissue due to impaired microfibril formation but also in dysfunctional TGF-β signaling [4,5].

The diagnosis of MFS depends on tabulating major and minor manifestations in different body systems according to the revised Ghent nosology of 2010, which heavily weights cardiovascular manifestations of MFS and results of *FBN1* studies [6]. Signs and symptoms of MFS vary widely in severity, time of onset and rate of progression. Cardinal manifestations involve the ocular, skeletal and cardiovascular systems. The most consequential morbidity and mortality in MFS relate to the degree of cardiovascular involvement, with dilation of the aorta at the level of the sinuses of Valsalva predisposing to aortic aneurysm; the risk of dissection and rupture lessens life expectancy. In addition, cardiac involvement with severe mitral- and/or aortic-valve insufficiency, with blood regurgitation, predisposes to left ventricular dysfunction and occasionally to heart failure [1,2,3,4,6].

Recent studies using advanced echocardiography and cardiac magnetic resonance imaging (CMR) have reported evidence of primary cardiomyopathy in adult and adolescent MFS patients [7,8,9,10,11] and myocardial fibrosis has been demonstrated histologically in mice with MFS [12,13].

Routine modalities for monitoring cardiovascular involvement in MFS patients are mainly imaging-based and are primarily focusing on vascular issues. Factors reportedly correlated with aortic aneurysm formation or dissection in MFS include serum concentrations of species involved in collagen metabolism, like TGF-β, MMP-3 and lysyl oxidase [14,15,16,17,18,19,20,21].

Biomarkers for the evaluation of the risk of the development of *myocardial* abnormalities are not reported in this patient group. In biventricular heart failure patients and in patients with congenital heart disease (CHD), candidate biomarkers derived from the metabolism of amino acids and lipids indicate not only general chronic low-level inflammation, oxidative stress and nitric oxide (NO) pathway alterations but also subtle myocardial dysfunction [22,23,24,25,26]. Thus, the study of amino acids and lipid metabolism offers promise, focusing on potential mechanisms of subtle alteration of myocardial function in patients with MFS.

Our objective, therefore, was to investigate amino-acid and lipid metabolism in adult MFS patients, seeking biomarker patterns that might complement imaging studies as monitoring tools in early cardiovascular risk assessment. Using a combined imaging and targeted metabolomics approach, we hypothesized to find signs of incipient cardiomyopathy as reflected by altered levels of selected metabolism-derived analytes reflecting activated proinflammatory processes and signaling pathways and an altered energy state as major mechanisms in the development of myocardial fibrosis.

## 2. Results

After applying all inclusion and exclusion criteria to the 40 patients who are actively managed in the aortopathy clinic of the Department of Pediatrics III (Pediatric Cardiology), Medical University of Innsbruck, Austria, 24 patients with confirmed MFS were eligible for the study. Four met the Ghent criteria for diagnosing MFS exclusively on clinical grounds. The other 20 had undergone genetic testing that found an *FBN1* mutation (Table 1).

**Figure 1:**
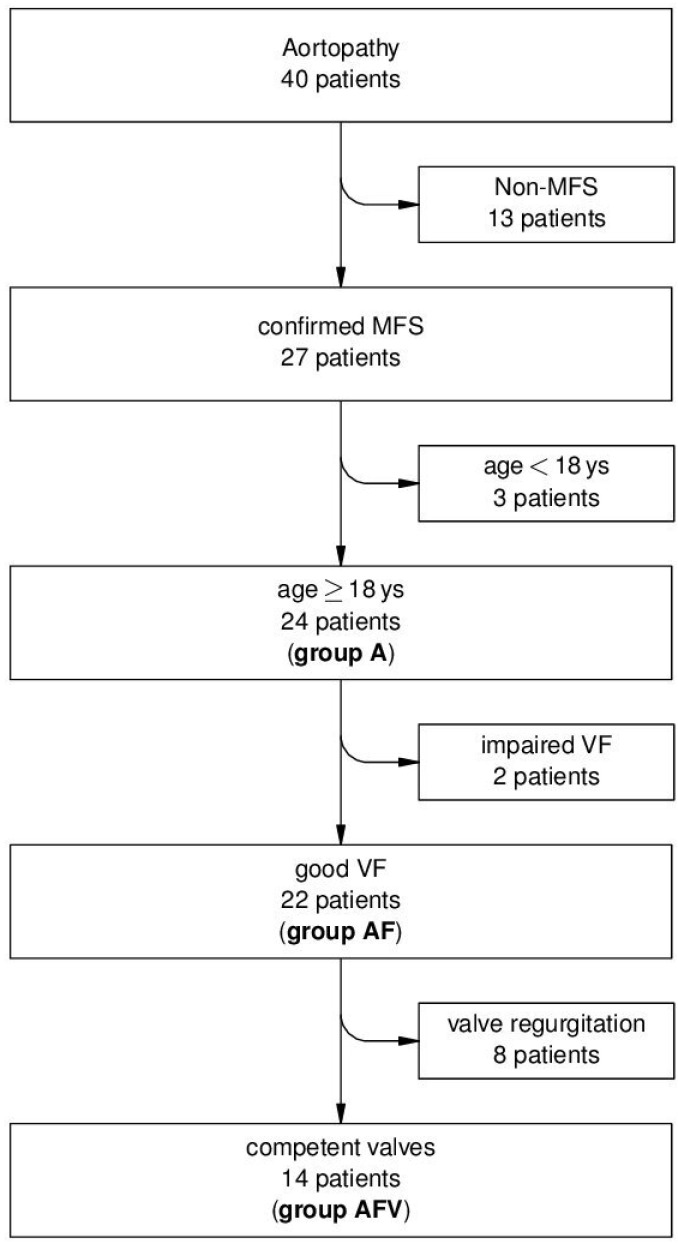
Patient recruitment and group assignment. MFS, Marfan syndrome; VF, ventricular function. *group A,* criterion age (>18 years), *group AF*, criteria age (≥18 years) and function (good systolic ventricular function); *group AFV*, criteria age (≥18 years), function (good systolic ventricular function: EF ≥ 55%, FS ≥ 27%), valve (competent valves).

Within the frame of the analysis, besides the *total group* of 24 patients (≥18 years, confirmed MFS) (group A, criterion age (≥18 years)), additionally we focused on the subordinate *group AF*, criteria age (≥18 years) and function (good systolic ventricular function) (n = 22) and on the subordinate *group AFV*, criteria age (≥18 years), function (good systolic ventricular function: EF ≥ 55%, FS ≥ 27% as assessed by echocardiography and CMR [27,28] within the 12 months before initiation of this study), valve (competent valves) (n = 14). The three groups, thus, were *not* mutually exclusive. Figure 1 gives an overview of patient flow and subgroup assignment.

Table 2 lists clinical and imaging parameters of the subjects. Of note is that a high proportion of patients in all three groups had echocardiographically or CMR-assessed signs of impaired diastolic function [27,28]. A high proportion of patients in all three groups had undergone David surgery (group A: 12/24, AF: 12/22, AFV: 6/14), in which the aortic root is replaced by a graft (valve-sparing approach); a smaller proportion had undergone Bentall surgery (group A: 3/24; AF: 3/22; AFV: 2/14), with composite graft replacement of both the aortic root and the aortic valve. Of the total 15 patients who had undergone aortic root surgery, the median time after surgery at the time of examination was 7 years (min 2, max 18 years).

Table 3 presents values for routine analytes in patients and controls. Note that compared with controls, patients in all three groups had slightly elevated serum levels of N-terminal prohormone of brain natriuretic peptide (NT-proBNP) (laboratory-set reference range for adults, 0–86 pg/mL).

### 2.1. Amino Acids

**Figure 2:**
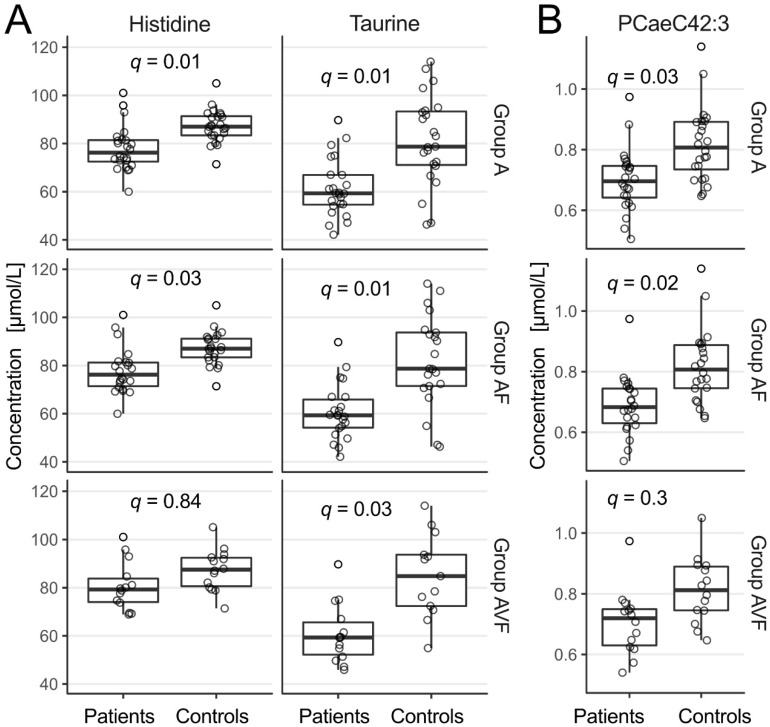
Box-and-whisker plots of serum concentrations of the amino acids histidine and taurine (**A**) and of the serum concentration of the phosphatidylcholine PCaeC42.3 (**B**), which differed significantly between Marfan patients and controls. The boxes show the 1st (Q1) and 3rd quartile (Q3), the whiskers the minimum and the maximum. Jitter plot of all data points overlaid on boxplot of the data. Black points represent outlying values as identified by the interquartile range (IQR) rule (values smaller than (Q1 − 1.5 × IQR) or values larger than (Q3 + 1.5 × IQR)). Group A, all patients; group AF, patients with preserved systolic ventricular function; group AVF, patients with preserved systolic ventricular function and without major valve regurgitation. *q*, FDR-adjusted *p*-values.

We assessed 42 amino acids and biogenic amines, including their derivatives. Of those 42 analytes, 27 passed initial quality control (Table S1). In patients, serum concentrations of taurine (False discovery rate (FDR)-adjusted p-values (*q*-values): group A: *q* = 0.01; group AF: *q* = 0.01 group AFV: *q* = 0.03) and histidine (group A: *q* = 0.01; group AF: *q* = 0.03) were significantly lower than in controls (Figure 2a), while sums and ratios did not differ.

### 2.2. Phospholipids and Acylcarnitines

We assessed 14 lyso-phosphatidylcholine (PC) (10/14 above limit of detection (LOD)), 76 PC (69/76 above LOD) and 15 sphingomyelin (SM) (all above LOD). Among the analytes passing initial quality control, the serum concentration of one phospholipid, PCaeC42:3, was significantly lower in patients than in controls (groups A: *q* = 0.03; group AF: *q* = 0.02; Table S1, Figure 2b). The concentrations of all other PC or SM as well as their summed concentrations did not differ between patients and controls. Nor did summed concentrations of PC-specific subgroups with regard to their cumulative content of double bonds (saturated, mono- and polyunsaturated PC) differ between patients and controls. We assessed 40 acylcarnitines, of which 5 passed initial quality control. Neither serum concentrations of the individual acylcarnitines nor their total concentration differed between patients and controls.

Table S1 gives concentrations and statistical results for all metabolites determined.

### 2.3. Univariate Correlation of Routine Biochemical and Clinical Findings with Metabolomic Parameters

Among routine biochemical analytes in patients, values for total cholesterol exhibited significant positive correlations with PCaeC38:3 (group A: *q* = 0.009, *r* = 0.78; group AF: *q* = 0.018, *r* = 0.79). High-density lipoprotein cholesterol was significantly and positively correlated with PCaaC32:0 (group A: *q* = 0.003, *r* = 0.75; group AFV: *q* = 0.014, *r* = 0.91) and PCaeC32:2 (group AFV: *q* = 0.015, *r* = 0.9). No significant correlations existed between values for routine biochemical analytes and amino acids or their sums or ratios. In controls, among routine analytes only C-reactive protein (CRP) and NT-proBNP were determined, which—as in patients—were not significantly correlated with any of the metabolomics parameters.

Because serum levels of NT-proBNP were significantly higher in patients than in controls, we assessed correlation between NT-proBNP level and aortic diameter at the level of the aortic root, or at the level of the ascending aorta (expressed as z-scores). While no correlation with aortic root diameter existed (group A: *q* = 0.17, *r* = 0.4; group AF: *q* = 0.07, *r* = 0.57; group AFV: *q* = 0.6, *r* = −0.3), in all three groups a significant positive correlation existed between serum NT-proBNP level and ascending aorta diameter (group A: *q* = 0.007, *r* = 0.69; group AF: *q =* 0.002, PCC 0.79; group AFV: *q* = 0.02, PCC 0.8).

### 2.4. Multiple Regression Analysis of Metabolomics Parameters with Respect to Group Assignment

The serum concentrations of the analytes that differed between groups (taurine, histidine and PCaeC42:3) were significantly correlated with patient group assignment (A: *p* < 0.0001, *r*^2^ 0.53; AV: *p* < 0.0001, *r*^2^ 0.51; AVF: *p* = 0.0004, *r*^2^ 0.52). In addition, they were significantly associated with the z-score of the ascending aorta (A: *q* = 0.048, *r*^2^ −0.42; AV: *q* = 0.001, *r*^2^ 0.06) (Table S2a).

Vice versa, focusing on the laboratory variables which were different between patients and controls as response parameters, we found a significant correlation between the variables ‘evidence of diastolic dysfunction’, “history of aortic root surgery” and aortic root dimensions with the serum concentration of NT-proBNP (AV: *q* = 0.02, *r*^2^ 0.2) and taurine (A: *q* = 0.002, *r*^2^ 0.83; AV: *q* = 0.002, *r*^2^ 0.96) (Table S2b).

## 3. Discussion

### 3.1. Main Findings

With a combined imaging and serum metabolomics approach in a group of adult MFS patients without major valve disease and heterogeneous in terms of presence or absence of prior aortic root surgery and type of surgical approach we found that a high proportion of patients revealed altered diastolic myocardial function and that their serum levels of histidine, taurine and PCaeC42:3—all of the three analytes reportedly involved in inflammation, oxidative stress, endothelial function processes—were lower than in controls.

### 3.2. Inflammation, Oxidative Stress, Endothelial Function

Inflammation, oxidative stress and altered endothelial function are known pathophysiological mechanisms for vascular abnormalities in patients with aortopathies. Being involved in signaling, thereby affecting synthesis of prostaglandins and leukotrienes and modulating inflammation, phospholipids are analytes interesting to focus on [29,30,31,32]. Using metabolomic approaches, Doppler et al. found levels of phospholipids that differed from those in controls in patients with bicuspid aortic valve-associated aneurysms and in patients with tricuspid aortic valve-associated aortic dissections, albeit in tissue rather than in serum [32]. This is in line with the drop in PCaeC42:3 we found: the etherlipid PCaeC42:3 is part of the external lipid bilayer of cells in all tissues, determining fluidity of cell membranes and is involved in signaling pathways of synthesis of prostaglandins and leukotrienes synthesis.

The drop in histidine serum levels we found points into the same direction of underlying (low-level) inflammation in our patients: histidine is decarboxylated to histamine by histidine decarboxylase [33]. This mechanism contributes to histamine release, a known triggering and maintaining analyte for inflammatory processes.

Inflammatory processes in patients with aortopathies, particularly MFS, are also reflected by other mechanisms like an altered protein-carbonyl content, higher numbers of T-cells and macrophages infiltrating the medial smooth muscle cell layer, increased serum concentrations of macrophage colony stimulating factor and an increased expression of class II major histocompatibility complex genes [16,17,18,19,20,34].

Inflammation is typically accompanied by features of oxidative stress and altered endothelial function, thus, e.g., alterations of methionine (Met)-sulfoxide (SO) levels and Met-SO/Met ratios reflecting the process of scavenging reactive species as protection against oxidative stress may have been likely to exhibit alterations [35]. However, in our study Met-SO was not above LOD in our measurements. Further, altered levels of immediate components of NO metabolic pathways, such as altered levels of arginine, citrulline, or methylarginines—e.g., asymmetric dimethylarginine may have been unmasked if we had had the chance to assess a larger patient group with less varying surgical treatment [21,36,37,38,39,40,41].

### 3.3. Myocardial Function

Of importance is that in patients with congestive heart failure decreased serum concentrations of histidine and taurine indicate not only oxidative stress, but also altered (myocardial) cell energy metabolism and elevated myocardial protein turnover in the presence of hyperreactive oxygen species [22,23,42]. Taurine abounds in myocardial tissue, where it has a major function in respiratory-chain regulation. The taurine-deficient heart suffers impaired respiratory-chain function and diminished mitochondrial long chain fatty acid uptake, as found in patients with congestive heart failure. Myocardial concentrations of taurine are altered in patients with dilated cardiomyopathy and many studies report that taurine supplementation beneficially affects myocardial function in patients with congestive heart failure [23,43].

Of interest is that serum values of taurine were decreased even in the very small AFV group (good ventricular function and competent valves), thus, corroborating imaging study suggestions that MFS patients tend to develop alterations of myocardial function and structure even in the absence systolic impairment and valvular regurgitation. Indeed, higher NT-proBNP levels, indicating deteriorating ventricular function, are reported in young adult patients with MFS [44]. A potential benefit of taurine supplementation in MFS patients might be worth considering [23,43].

It is not clear why the taurine serum concentration is decreased in MFS patients; we cannot exclude a reduced intestinal amino acid (esp. methionine) uptake. None of our patients had a dietary restriction nor was any of them vegetarian or vegan. Further studies alluding to intestinal uptake and, particularly in terms of taurine, also bile acid metabolism may be interesting. Another open question is whether our data indicate primary or secondary alterations of endothelial pathophysiology in general or at the cardiac level. MFS children and young adults have altered aortic flow patterns and differences in wall shear stress that are most pronounced in aortic root and the proximal parts of the distal aorta, segments where aortic dissection or rupture often originate [45].

The significant correlation of serum levels of histidine, taurine and PCaeC42:3 with the evidence of diastolic dysfunction and aortic root dimensions we found implies that these analytes indicate both vascular and myocardial alterations in MFS patients. One might attribute altered myocardial properties to hemodynamic abnormalities arising in altered aortic root geometry. Even more important will certainly be abnormalities in hemodynamics and myocardial function following aortic root surgery, since a high proportion of our patients had had such surgery. However, of note is that a CMR study in unoperated pediatric patients with MFS found aortic root dilation, valvular incompetence, mild biventricular systolic dysfunction and diffuse myocardial fibrosis [46]. Interestingly, those alterations were less pronounced in patients taking losartan. The presence and extent of myocardial fibrosis could not be assessed in our patients owing to our study setup, but the matter calls for investigation.

Our findings raise the suspicion that myocardial abnormalities in MFS arise not only from disordered extracellular matrix remodeling caused by intramyocardial fibrillin-1 deficiency and dysregulation of TGF-β with extracellular matrix remodeling but also from oxidative stress and altered endothelial function. To better understand the pathophysiology of subtle cardiomyopathy in the patient with MFS, closer attention to oxidative stress and endothelial function is required. Such a focus might also suggest therapeutic approaches: For example, NO pathway effects of losartan in MFS patients may result from the role of NO as a negative regulator of TGF-β [47].

### 3.4. Limitations, Confounders

Because MFS patients are few and the cohorts that can be assembled are heterogeneous, our study groups were small. Further, we did not have the chance replicate our examinations. These points limit generalization from our findings, especially with regard to our subanalysis in terms of previously undergone surgery and type of surgery. Larger studies in patient groups more homogeneous in respect of surgery are desirable, particularly in unoperated individuals. In addition, comparison of serum amino acid levels between MFS patients with and patients without demonstrably impaired diastolic function or with signs of fibrosis (as on CMR study) would be desirable. That said, it is important to mention that the assessment of diastolic dysfunction in our setting imposes a limitation per se: we did not assess tissue velocities or left atrial volumes, which would be mandatory for the detection of patients with grade II diastolic dysfunction and pseudonormalization of their E/A ratio. Importantly, only two of the three patients with increased left ventricular enddiastolic volume (LVEDV) showed frank grade III diastolic dysfunction. In the third patient diastolic dysfunction was thus solely picked up by means of CMR, underlining the role of CMR volumetry in this patient group. It is hence assumable that there may be more among our patients who would be classified with diastolic dysfunction, which we now missed [27,28].

In addition, limiting may be the targeted metabolomic approach using a commercial kit, as metabolites not covered by the kit may be important. However, in terms of project implementation, our choice seemed reasonable. In addition, as we did not know on exactly which distinct single metabolites to focus, we elected the relatively broad (albeit targeted) approach provided by the kit that we used. In concert with our metabolomics studies in patients with complex CHD, the results of our current study and the finding that the metabolomics approach is feasible in adult MFS patients may help configure a metabolomics approach methodologically adapted to requirements for patients with CHD and connective tissue disorders [24,25,26].

The concentration of numerous analytes detectable with the kit that we used was around or just below LOD, including concentrations of analytes that are of particular interest in terms of oxidative stress (e.g., methionine sulfoxide) or myocardial energy metabolism (e.g., acylcarnitines). Studies in healthy individuals have determined that up to a third of targeted analytes are undetectable and therefore excluded from analyses, although for some tissues or pathological conditions analyte levels may rise to detectability [48]. Hence, contributing to ideas to optimize the kit’s use in patients with CHD or connective tissue disorders, the LOD might ideally be adapted.

We analyzed serum samples, thereby precluding direct comparison between our results and those of studies that used tissue samples. We acknowledge that having assayed serum rather than vascular or myocardial tissue, especially the point of altered myocardial energy metabolism maintains speculative and that for definite confirmation we would need to examine myocardial tissue. Moreover, we cannot rule out certain system-driven aspects of disease pathogenesis: Unknown confounders that affect metabolic profiles might be the true basis for the observed differences. Moreover, differences body composition or lifestyle parameters may have influenced our results to an unknown degree. We strove to lessen the likelihood of such errors by following a strict inclusion and exclusion protocol, especially with regard to (known) comorbidities and medication and diet (vegetarian/vegan vs. non-vegetarian/vegan). However, the socioeconomic status might affect further aspects like physical activity and diet (beyond vegetarian/vegan or not). To lessen these confounders, it would have been favorable to include probands from different socioeconomic status (in our case a high percentage of control probands were staff of the University). Lastly, we cannot exclude that differences in body height, weight (BMI), or kidney function between patients and controls confounded our findings; to match probands with controls for those values might be valuable.

## 4. Materials and Methods

### 4.1. Study Design, Inclusion and Exclusion Criteria

This study is an observational cohort study. At the Department of Pediatrics III (Pediatric Cardiology), Medical University of Innsbruck, Austria, between January 2019 and January 2020, we prospectively examined a cohort of adult patients with MFS (heterogenous in respect of history of prior aortic root surgery as well as of type of surgery) and a second cohort of healthy biventricular controls matched for age and sex, albeit not for height. Controls were randomly recruited among staff of the Departments of Pediatrics and Pediatric Cardiology, Medical University of Innsbruck, Austria.

Eligibility criteria for all subjects were written informed consent; age at testing of ≥18 years; overnight (at least 5 h) fasting before blood sampling; and morning blood sampling. Additional criteria were for *patients* a clinically or genetically confirmed diagnosis of MFS and for *controls* both a biventricular heart without any structural or functional abnormality and the absence of systemic—including cardiovascular—disease.

Exclusion criteria were ingestion of any medication directly affecting the metabolic state (e.g., cholesterol-lowering agents) or hemodynamic state (e.g., diuretics), excepting beta-blockers and angiotensin II receptor antagonists in MFS patients; atrial or ventricular arrhythmia as assessed by 12-channel electrocardiogram; coronary artery disease (history of myocardial infarction, myocardial revascularization, percutaneous coronary intervention, or coronary artery bypass surgery); any metabolic disease, such as diabetes mellitus; malignancy or other disease leading to cachexia; liver or renal disease; inflammatory disease such as acute or chronic infection; a myeloproliferative disorder; pregnancy or lactation; multiple organ failure; malnourishment; and mental handicap not allowing for valid consent to study participation.

### 4.2. Outcome

Outcome criteria were myocardial function [27,28] (sustained vs. impaired diastolic function as assessed by means of cardiac imaging and NT-proBNP levels) and the compared to healthy controls different pattern of serum levels of amino acids, acylcarnitines and phospholipids suggesting low-level inflammation or altered myocardial function.

### 4.3. Routine Examination

Age, sex, weight, body mass index, vital parameters, cardiac risk factors, history of cardiac disease and history and type of cardiovascular surgery, cardiac medication, routine hematological-study biomarkers and biochemical biomarker profiles were assessed during an outpatient-clinic visit. Fasting participants underwent phlebotomy while recumbent, followed by echocardiography using traditional parameters of systolic (left ventricular shortening fraction (SF), ejection fraction (EF)) and diastolic (E/A ratio) function and cavity dimension (Acuson S2000, Siemens Healthineers, Erlangen, Germany). For CMR studies, a Siemens Magnetom Avanto (Siemens Healthineers, Erlangen, Germany) fit (1.5 Tesla) was used and aortic root and ascending dimension were assessed, as were systolic and diastolic left ventricular function by volumetry; owing to study design, information on T1 or late gadolinium enhancement was not collected. Impaired systolic left ventricular function was defined as EF < 55% and FS < 27%, as assessed by echocardiography and CMR within the 12 months before initiation of this study.

Impaired diastolic function was defined as a mitral E/A ratio ≤0.8 or ≥2 as assessed by echocardiography [27] and as an increased LVEDV according to the recently updated published reference values [28].

#### Sample Preparation and Metabolomics Analysis

Blood studies required samples 0.5 mL larger than those for routine assessments to allow determinations of concentrations of acylcarnitine-, phospholipid- and amino acid-metabolism analytes.

Within 20 min after blood draw each blood sample was directly drawn into a tube containing a clotting activator and centrifuged (15 °C, 10 min, 2500× *g*) to separate serum. Serum aliquots of 0.1 mL were immediately frozen and stored at −80 °C for further analyses. Frozen samples were transported on dry ice to the analyzing laboratory. Analyses were performed in batches of 80 samples.

Before batch analysis, frozen samples were thawed on ice, then centrifuged; the supernatant was subjected to further analyses [24,25,49]. Endogenous metabolites were analyzed with a targeted quantitative and quality-controlled metabolomics approach using the AbsoluteIDQ^®^ p180 Kit (BIOCRATES Life Science AG, Innsbruck, Austria). We selected this assay as it is validated and well described in literature and, as it allows comprehensive identification and quantitation of a variety of endogenous metabolites derived from the amino acid- and lipid metabolism, we aimed to focus on (i.e., 42 amino acids and biogenic amines, 40 acylcarnitines and 105 PC and SM, hexose).

Targeted analysis for these metabolites applied flow injection analysis-tandem mass spectrometry (FIA-MS/MS), as well as liquid-chromatography-tandem mass spectrometry (LC-MS/MS) with multiple reaction monitoring in positive electrospray ionization mode. Quantitation was achieved with internal standards.

The AbsoluteIDQ^®^ p180 kit, a 96-well plate format assay, was applied as described in the manufacturer’s instructions. Sample aliquots (10 µL) were pipetted onto filter spots suspended in the wells of a 96-well filter plate. The filter plate was fixed on top of a deep-well plate that later served to receive extracts; the ensemble was termed a combi-plate. After brief drying under a nitrogen stream, 50 µL of a 5% phenylisothiocyanate solution was added to form amino-acid derivatives. After 20 min of shaking and nitrogen drying, 300 µL of 5 mM ammonium acetate in methanol was added to the wells. After 30 min of incubation, the combi-plate was centrifuged to transfer the extracts into the deep-well plate, which then was detached from the upper filter plate. Samples for FIA-MS/MS were prepared by mixing 10 µL of the extracts with 40 µL of 5 mm ammonium acetate in methanol. For LC-MS/MS, 30 µL of the extracts were diluted with 30 µL of water.

Quantitative analysis was performed on a mass spectrometric system consisting of a 1100 series high-performance liquid chromatography pump (Agilent, Waldbronn, Germany), a CTC-PAL autosampler (CTC Analytics AG, Zwingen, Switzerland) and a SCIEX 4000 QTRAP^®^ instrument (SCIEX, Toronto, ON, Canada). The injection volume was 20 µL. For FIA-MS/MS, the flow rate was set to 30 µL/min. For LC-MS/MS analysis, separations were accomplished on an Eclipse XBD-C18 column (3.5 µm, 3.0 × 100 mm, Agilent) using a five-minute gradient of 0–95% acetonitrile in aqueous 0.2% formic acid solution. The flow rate was set to 500 µL/min and the column temperature was kept at 50 °C.

Metabolite concentrations (µM) were calculated by the MetIDQ™ software package version 7.11.5 (Biocrates, Innsbruck, Austria) or Analyst 1.5 software version 1.6.3 (SCIEX, Redwood City, CA, USA).

### 4.4. Statistics

Metabolites that showed insufficient measurement stability for the quality control samples (relative standard deviation of metabolite concentrations >30%) or metabolites that did not exceed the LOD in at least 50% of samples were identified and excluded from further evaluation. Quantitative information for 127 metabolites was subjected to univariate statistical analysis. Normality was tested using the Shapiro–Wilk test. Homogeneity of variance was tested using Levene’s test or the Kruskal–Wallis test as appropriate. Student’s *t*-testing or Mann–Whitney U testing with a FDR correction according to Benjamini and Hochberg identified significant metabolite differences between patients and controls. Statistically significant differences between patients and controls were characterized by FDR-adjusted *p*-values (*q*-values) < 0.05. Analytes for which values differed between patients and controls then were assessed via multiple regression analysis to determine the analyte’s ability to predict group assignment or other response variables (evidence of diastolic dysfunction, history of aortic root surgery, aortic diameter). Correlations between analyte concentrations (and their sums and ratios, as specified in the Results section) and routine analytes (and clinical variables) of the participants were evaluated by Pearson’s correlation coefficient *r*. The significance of the correlation was calculated from *r* and the degrees of freedom (a variable dependent on sample number) to interpret correlations. The resulting *p*-value was FDR-adjusted for multiple testing. Correlations with FDR-adjusted *p*-values < 0.05 and *r* > 0.5 (or <−0.5) were considered statistically significant. Statistical analysis and data visualization was performed using R version 4.0.2 (R Core Team, 2020).

### 4.5. Pathway Analysis

Information on analytes and the pathways in which they are involved is mainly based on https://www.metaboanalyst.ca/ (Accessed on 2 February 2021) and on the Kyoto Encyclopedia of Genes and Genomes.

## 5. Conclusions

Our study confirms reported findings of impaired diastolic function in a high proportion of patients with MFS. It also reveals new information on possible concomitant alterations or mechanisms: Changes in serum levels of isolated metabolism derived analytes link MFS pathophysiology with altered inflammation, oxidative stress and subtle changes in myocardial functional and structural properties, as found in patients with congestive heart failure. Markers identified through mass spectrometry-based extended investigation particularly of the amino acid metabolism might complement traditional diagnostic imaging tools and traditional laboratory biomarker determinations, yielding a better understanding of the development of myocardial deterioration in MFS patients. Particularly taurine may play a future role in the detection of possible targets for therapeutic approaches to mitigate inflammation in propagating ventricular deterioration in MFS patients.

## Figures and Tables

**Table 1:** Genetic variants of MFS patients included. #, number.

Patient #	Variant of *FBN1* Point Mutation	Protein Phenotype	Predicted Protein Phenotype	Frequency in UMD-FBN1 Database	Pathogenicity
1	c.1754G > A	p.G585E	Missense, loss of function	0	class 4
2	c.6453C > A	p.C2151Ter	Stop codon at p.2151	1	class 5
3	c.508delT	p.Y170Tfs*20	Frameshift with stop codon at p.189	1	class 5
4	c.508delT	p.Y170Tfs*20	Frameshift with stop codon at p.189	1	class 5
5	c.7801C > T	p.Q2601*	Missense, loss of function	2	class 5
6	c.4172G > T	p.C1391F	Disulfide bond interruption	1	class 5
7					
8					
9	c.732dupT	p.Q245STerfs*5	Frameshift with stop codon at p.248	0	class 4
10	c.7801C > T	p.Q2601*	Missense, loss of function	2	class 5
11	c.7801C > T	p.Q2601*	Missense, loss of function	2	class 5
12	IVS14-2A > G	Intron 14 SNP	Invariant AG acceptor splice site mutation	0	class 5
13	c.6453C > A	p.C2151Ter	Stop codon at p.2151	1	class 5
14	IVS45+3insCC	Intron 45 insert	Loss of exon 45	0	class 4
15	c.6313+5G > A	Intron 51 SNP	Splice error, exon 50	3	class 4
16	c.5840G > T	p.C1947F	Disulfide bond interruption	1	class 5
17	c.6675T > A	p.Y2225*	Stop codon at p.2225	0	class 4
18	c.2638G > A	p.G880S	Missense, loss of function	12	class 4
19	c.732dupT	p.Q245STerfs*5	Frameshift with stop codon at p.248	0	class 4
20	c.732dupT	p.Q245STerfs*5	Frameshift with stop codon at p.248	0	class 4
21	c.6313+5G > A	Intron 51 SNP	Splice error, exon 50	3	class 4
22	c.4202delC	p.T1402Terfs*11	Frameshift with stop codon at p.1405	1	class 4
23					
24	c.4816G > A	p.D1606N	Splice error, exon 39	3	class 4

**Table 2:** Participants’ clinical characteristics.

-	-	Group A			Group AF			Group AFV		
		Patients	Controls	*p*	Patients	Controls	*p*	Patients	Controls	*p*
Total [n]		24	24		22	22		14	14	
Female sex [n]		13	13		12	12		7	7	
Age [ys]		37 ± 13	37 ± 13	0.9	38 ± 13	38 ± 13	0.9	36 ± 11	36 ± 11	0.9
Bodyweight [kg]		77 ± 13	74 ± 15	0.4	75 ± 13	74 ± 16	0.7	79 ± 12	72 ± 14	0.1
Height [cm]		185 ± 9	174 ± 8	0.00	184 ± 9	174 ± 8	0.00	187 ± 9	174 ± 9	0.00
BMI [kg/m²]		22 ± 3	24 ± 4	0.1	22 ± 3	24 ± 4	0.08	22 ± 3	23 ± 4	0.6
BP systolic [mm Hg]		115 ± 10	122 ± 9	0.02	115 ± 10	121 ± 9	0.06	115 ± 11	118 ± 9	0.5
BP diastolic [mm Hg]		81 ± 8	79 ± 8	0.3	81 ± 7	78 ± 8	0.2	80 ± 8	78 ± 9	0.6
HR at rest [1/min]		57 ± 9			57 ± 10			56 ± 8		
SpO_2_ at rest [%]		99 ± 1			99 ± 1			99 ± 1		
Cardiac manif. [n]	Aortic dilation	22			22			13		
	AR	2			2			2		
	MV prolapse	17			15			10		
	MR	1			1			1		
Cardiac procedure [n]	Bentall	3			3			2		
	David	12			12			6		
	Stent graft thor. aorta	1			1			1		
	Repl. thor. aorta	1			1			0		
	MV reconstruction	1			1			0		
	PM implantation	1			1			1		
EC manifestation [n]	Facial features									
	Dolichocephaly	15			13			8		
	Enophthalmos	3			3			2		
	Retrognathia	6			6			5		
	High arched palate	20			18			11		
	Malocclusion	1			1			1		
	Crowding of teeth	2			2			0		
	Skeleton									
	Arachnodactyly	21			19			11		
	Steinberg positive	20			18			11		
	Murdoch positive	19			18			11		
	Joint hypermobility	10			10			7		
	Pectus carinatum	7			6			3		
	Pectus excavatum	8			7			5		
	Scoliosis	17			16			10		
	Kyphosis	6			6			4		
	Spinal disc herniation	1			1			1		
	Protrusio acetabuli	9			9			5		
	Coxarthrosis	2			2			1		
	Hindfoot deformity	11			10			6		
	Hallux valgus	6			6			3		
	Skin									
	Striae	21			21			13		
	Eyes									
	Myopia	11			11			8		
	Astigmatismus	3			3			2		
	Cataracta senilis	3			3			2		
	Strabismus conv.	1			1			0		
	Subluxatio lentis	4			3			3		
	Ectopia lentis	6			6			4		
	Iridodonesis	3			3			1		
	Vitreous prolapse	1			1			1		
	Ablatio retinae	2			2			1		
	Chest									
	Pneumothorax	2			2			1		
	CNS									
	Tarlov cyst	1			1			1		
EC procedures [n]	Hip prosthesis	2			2			1		
	Scoliosis surgery	2			1			1		
	Pigeon chest correction	1			1			1		
	Pectus excavatus surg.	2			2			1		
	Lens enucleation	1			1			0		
	Lentectomie	1			1			1		
	Phacoemulsification	2			2			1		
Further disease [n]	Morbus Scheuermann	1			1			1		
	Spondyloarthrosis	1			1			1		
	Knee valgus	3			3			2		
	Baker cyst	1			1			1		
	Metatarsalgia	2			2			2		
	Lyme arthritis	1			1			1		
	Varicose veins	1			1			0		
	Cerebral infarction	2			2			1		
	Hyperopia	1			1			1		
	Exopthalmus	1			1			1		
Further procedure [n]	Cesarean section	2			2			2		
	Cruciate ligament surg.	2			2			2		
	Meniscus surg.	1			1			1		
	Tooth regulation	6			6			4		
	Vein stripping	1			1			0		
	Orchidopexy	1			1			1		
Dietary intake [n]		0			0			0		
*Medication*										
ACE inhibitor [n]		1			1			1		
Betablocker [n]		17			16			8		
Aldosterone ant. [n]										
AT-II rec. ant. [n]		15			15			8		
Statin		2			2			0		
*Echo/CMR*										
EF < 55% or FS < 27%		2			0			0		
DD *		10			8			5		
≤mild AVR [n]		14			14			14		
≤mild AR [n]		14			14			14		
Aortic root [mm]		33 ± 5.7			37 ± 5.1			35 ± 4		
Aortic root [z-score]		2.1 ± 1.7			2.1 ± 1.7			1.1 ± 1.3		
Asc. aorta [mm]		30 ± 5.8			30 ± 5.6			30 ± 4.6		
Asc. aorta [z-score]		1.4 ± 1.9			1.7 ± 1.8			1.4 ± 1.7		
*ECG*										
Incomplete RBBB [n]		9			9			7		
AVB grade I [n]		1			1			1		
QTc prolongation [n]		2			1			1		

Participants’ clinical characteristics. Values are given as mean ± standard deviation. *p* < 0.05 was considered statistically significant. Group A, all patients; group AF, patients with preserved systolic ventricular function; group AFV, patients with preserved systolic ventricular function and without major valve regurgitation. ACE, angiotensin converting enzyme; ant., antagonist; asc., ascending; AR, aortic regurgitation; AT, angiotensin; AVB, atrioventricular block; AVR, atrioventricular valve regurgitation; BP, blood pressure; CMR, cardiac magnetic resonance; CNS, central nervous system; DD, diastolic dysfunction (* as assessed by E/A inflow and LVEDV); echo, echocardiography; EC, extracardiac; ECG, electrocardiogram; EF, ejection fraction; FS, fractional shortening; HR, heart rate; manif., manifestation; MFS, Marfan syndrome; MV, mitral valve; PM, pacemaker; QTc, corrected QT time; RBBB, right bundle branch block; rec., receptor; SpO_2_, pulsoxymetric oxygen saturation; surg., surgery; thor., thoracic.

**Table 3:** Values of routine analytes.

Laboratory Value	Group A		*p*	Group AF		*p*	Group AFV		*p*
	Patients	Controls		Patients	Controls		Patients	Controls	
CRP [mg/dL]	0.29 ± 0.47	0.14 ± 0.19	0.159	0.35 ± 0.5	0.15 ± 0.19	0.215	0.44 ± 0.6	0.08 ± 0.07	0.08
NT-proBNP [pg/mL]	144 ± 103	37.4 ± 30.9	0.00	156.9 ± 107.4	24.9 ± 31.3	0.00	153.5 ± 99.1	30.5 ± 20.6	0.002
Total cholesterol [mg/dL]	192 ± 31			192.5 ± 32			195.9 ± 27.2		
HDL-C [mg/dL]	55 ± 14			54.5 ± 14.2			54.43 ± 14.9		
Non-HDL-C [mg/dL]	137 ± 32			138 ± 32.7			141.5 ± 22.4		
Triglycerides [mg/dL]	129 ± 54			129.8 ± 55.7			132.6 ± 52.3		
Total protein [g/dL]	7.2 ± 0.3			7.2 ± 0.3			7.1 ± 0.38		
Uric acid [mg/dL]	5.1 ± 1.1			5 ± 1.2			5.2 ± 1.0		
Urea [mg/dL]	29.0 ±6.7			28.8 ± 6.9			28.0 ± 6.5		
Creatinine [mg/dL]	0.7 ± 0.1			0.7 ± 0.1			0.7 ± 0.17		
Total bilirubin [mg/dL]	0.67 ± 0.5			0.6 ± 0.5			0.7 ± 0.6		
AST [U/L]	20.9 ± 5.6			21.3 ± 5.6			20.6 ± 6.8		
ALT [U/L]	19.8 ± 10.2			20 ± 10.8			21.9 ± 12.8		
gGT [U/L]	28.1 ± 26.7			28.9 ± 28.3			26.86 ± 19.9		
Alkaline phosphatase [U/L]	62.9 ± 15.3			64.4 ± 15			62.71 ± 12.6		
Leucocytes [1/nL]	5.8 ± 1.2			5.8 ± 1.2			5.6 ± 1.3		
Thrombocytes [1000/nL]	253 ± 64			258.3 ± 64.2			229.2 ± 44.4		
Hemoglobin [g/dL]	140.9 ± 11.3			141.2 ± 11.6			143 ± 9.6		
Hematocrit [%]	0.4 ± 0.03			0.4 ± 0.03			0.4 ± 0.03		

Values of routine analytes: Comparison of patients with controls. Values are given as mean ± standard deviation. *p* < 0.05 was considered statistically significant. Group A, all patients; group AF, patients with preserved systolic ventricular function; group AFV, patients with preserved systolic ventricular function and without major valve regurgitation. ALT, alanine aminotransferase; AST, aspartate aminotransferase; CRP, C-reactive protein; dL, deciliter; g, gram; gGT, gamma glutamyl transferase; HDL-C, high density lipoprotein-cholesterol; NT-proBNP, N-terminal prohormone of brain natriuretic peptide; U, unit.

## Data Availability

The datasets generated and analyzed during the current study are available upon request with appropriate IRB approval due to analyzing researcher’s policy.

## Supplementary Materials

The following are available online at https://www.mdpi.com/article/10.3390/metabo11120805/s1, Table S1 (a–c): Values of amino acids, of biogenic amines and their derivatives, and of selected sums and ratios for all three groups, Table S2: (a) Multivariate regression analysis, clinical and imaging parameters as out-come variables, (b): Multivariate regression analysis, biochemical parameters as outcome varia-bles.
