# Supplementary material for: Amino Acid and Phospholipid Metabolism as an Indicator of Inflammation and Subtle Cardiomyopathy in Patients with Marfan Syndrome
Source: Metabolites. 2021 Nov 27;11(12):805. doi: 10.3390/metabo11120805 (PMC8707072; doi:10.3390/metabo11120805)
Supplement: Supplementary file 1 [file metabolites-11-00805-s001.zip › metabolites-1462595-SI.pdf]

Supplemental tables

Table S1 a-c. Values of amino acids, of biogenic amines and their derivatives, and of selected sums and ratios for all three groups.

Metabolite concentrations ([µmol/l]) for all analytes with selected sums and ratios, grouped by Marfan patients *vs*. controls. Values are given as mean ± standard deviation. False discovery rate-corrected p (=q) < 0.05 was considered statistically significant. Group A, all patients; group AF, patients with preserved systolic ventricular function; group AFV, patients with preserved systolic ventricular function and without major valve regurgitation. FC, fold change (Marfan patients *vs*. controls); HMBD ID, Human Metabolome Database identification; AA, amino acids; AAA, aromatic amino acids; Ac-Orn, acethylornithine; ADMA, asymmetric dimethylarginine; alpha-AAA, alpha aminoadipic acid; Arg, arginine; BCAA, branched-chain amino acids; lysoPC, lysophophatidylcholine; orn, ornithine; PC, phosphatidylcholine; SM, sphingomyelin; ↑, statistically significant higher serum concentration in Marfan patients than in controls; ↓, statistically significant lower serum concentration in Marfan patients than in controls. 'Fischer ratio' is the ratio of BCAA/AAA.

Table S1 a. Values of amino acids, of biogenic amines and their derivatives, and of selected sums and ratios – all patients (group A).

Metabolite	HMBD ID	Patients	Controls	p	q	FC
ADMA	HMDB01539	0.54+-0.12	0.48+-0.13	0.14	0.56	1.11
Alanine	HMDB00161<br>HMDB01310	390+-90	400+-110	0.72	0.90	0.98
Arginine	HMDB00517<br>HMDB03416	118+-14	120+-20	0.64	0.85	0.98
Asparagine	HMDB00168<br>HMDB003378	48+-10	48+-8	0.90	0.96	0.99
Aspartic acid	HMDB00191<br>HMDB06483	19+-3	20+-4	0.40	0.70	0.96
C0	HMDB00062	36+-8	33+-5	0.16	0.57	1.08
C14:1	HMDB0002014	0.14+-0.05	0.13+-0.03	0.60	0.85	1.04
C18:1	HMDB0006351<br>HMDB0005065<br>HMDB06464<br>HMDB0013338	0.15+-0.05	0.14+-0.03	0.44	0.70	1.07
C18:2	HMDB0006469<br>HMDB0006461	0.053+-<br>0.019	0.049+-<br>0.017	0.43	0.70	1.08
C2	HMDB00201	7+-3	6.4+-1.6	0.61	0.85	1.05
Citrulline	HMDB00904	30+-9	32+-7	0.47	0.72	0.95
Creatinine	HMDB0000562	72+-16	83+-14	0.02	0.38	0.88
Glutamine	HMDB00641<br>HMDB03423	740+-90	730+-100	0.91	0.96	1.00
Glutamic acid	HMDB00148<br>HMDB03339	45+-22	32+-20	0.03	0.39	1.43
Glycine	HMDB00123	260+-50	310+-80	0.03	0.39	0.87
H1		3800+-<br>1300	3700+-500	0.87	0.95	1.01
**Histidine**	**HMDB00177**	**78+-9**	**87+-7**	**0.00**	**0.01**	**0.9**
Isoleucine	HMDB00172<br>HMDB0000557	70+-14	74+-13	0.38	0.70	0.95

Kynurenine	HMDB00684	4.4+-0.7	4.8+-0.8	0.11	0.56	0.92
Leucine	HMDB00687<br>HMDB0013773	100+-30	110+-30	0.22	0.62	0.90
Lysine	HMDB00182<br>HMDB03405	350+-40	320+-50	0.02	0.38	1.10
lysoPC a C16:0	HMDB10382<br>HMDB0061702	83+-13	83+-16	0.92	0.96	1.01
lysoPC a C16:1	HMDB0010383	3+-1.1	3.2+-0.8	0.42	0.70	0.93
lysoPC a C17:0	HMDB12108	7+-1	7.4+-1.6	0.27	0.67	0.94
lysoPC a C18:0	HMDB10384<br>HMDB0011128	28+-6	28+-5	0.95	0.96	1.00
lysoPC a C18:1	HMDB02815<br>HMDB0010385<br>HMDB0061701	23+-6	26+-8	0.14	0.56	0.89
lysoPC a C18:2	HMDB10386<br>HMDB0061700	32+-9	37+-15	0.19	0.57	0.87
lysoPC a C20:3	HMDB10394<br>HMDB0010393	3.1+-1	3+-0.8	0.89	0.96	1.01
lysoPC a C20:4	HMDB10395<br>HMDB0010396	8.4+-2	8+-3	0.64	0.85	1.04
lysoPC a C28:0	HMDB29206	0.5+-0.3	0.6+-0.3	0.23	0.62	0.81
lysoPC a C28:1	HMDB0029221	0.6+-0.4	0.8+-0.4	0.02	0.38	0.65
Methionine	HMDB00696	29+-4	31+-4	0.18	0.57	0.95
Ornithine	HMDB00214<br>HMDB03374	39+-17	46+-23	0.23	0.62	0.85
PC aa C28:1	HMDB0007867<br>HMDB0007899	2.9+-0.7	3.3+-0.7	0.12	0.56	0.90
PC aa C30:0	HMDB0007934<br>HMDB0007869<br>HMDB0007965	12+-3	12.1+-2.2	0.85	0.95	1.01
PC aa C30:2	HMDB0007999	1.21+-0.23	1.1+-0.3	0.19	0.57	1.08
PC aa C32:0	HMDB00564<br>HMDB0007871<br>HMDB0008031	14+-4	13+-3	0.39	0.70	1.06
PC aa C32:1	HMDB0007872<br>HMDB0007873<br>HMDB0008097<br>HMDB0007969	17+-10	17+-7	0.97	0.97	0.99
PC aa C32:2	HMDB0008002<br>HMDB0007874	4.6+-1.8	4.9+-1.7	0.46	0.71	0.92
PC aa C32:3	HMDB0007876	0.7+-0.16	0.73+-0.13	0.40	0.70	0.95
PC aa C34:1	HMDB0007971<br>HMDB08100<br>HMDB0008035<br>HMDB0007972<br>HMDB0008003	240+-60	250+-50	0.70	0.90	0.97
PC aa C34:2	HMDB07973<br>HMDB0008101<br>HMDB0008133<br>HMDB0008005<br>HMDB0008004	390+-50	390+-70	0.92	0.96	1.00

PC aa C34:3	HMDB08006<br>HMDB0007974<br>HMDB0007975	13+-4	15+-6	0.18	0.57	0.87
PC aa C34:4	HMDB0007883<br>HMDB0007976	1.9+-0.9	1.9+-0.7	0.87	0.95	0.98
PC aa C36:0	HMDB0008265<br>HMDB0008036<br>HMDB0008525<br>HMDB0007886	5.8+-1.2	5.9+-1.1	0.73	0.90	0.98
PC aa C36:1	HMDB08038<br>HMDB0008069<br>HMDB0007978<br>HMDB0008102	61+-14	65+-13	0.40	0.70	0.95
PC aa C36:2	HMDB08039<br>HMDB0008070<br>HMDB08135<br>HMDB0007979	230+-40	230+-40	0.94	0.96	1.00
PC aa C36:3	HMDB08105<br>HMDB0007980<br>HMDB0007981<br>HMDB0008040	133+-25	130+-30	0.82	0.95	0.99
PC aa C36:4	HMDB07982<br>HMDB0008107<br>HMDB0008138<br>HMDB0008234<br>HMDB0008429<br>HMDB0008170<br>HMDB0008138<br>HMDB0008042<br>HMDB0008203<br>HMDB0008106	170+-40	160+-30	0.12	0.56	1.10
PC aa C36:5	HMDB07984<br>HMDB0008015	20+-13	20+-12	0.85	0.95	0.97
PC aa C36:6	HMDB0008206<br>HMDB0007892	0.8+-0.4	0.9+-0.4	0.51	0.77	0.92
PC aa C38:0	HMDB0007893<br>HMDB0008528<br>HMDB0008043<br>HMDB0008755<br>HMDB0008267<br>HMDB0007985	3.4+-0.6	3.2+-0.7	0.42	0.70	1.05
PC aa C38:1	HMDB0007894<br>HMDB0008269<br>HMDB0007986<br>HMDB0008268<br>HMDB0008109<br>HMDB0008044	0.13+-0.18	0.23+-0.25	0.11	0.56	0.56
PC aa C38:3	HMDB0008020<br>HMDB0008046<br>HMDB0008047	50+-12	47+-11	0.30	0.70	1.07
PC aa C38:4	HMDB0008048<br>HMDB0008112<br>HMDB0008113<br>HMDB0007988	101+-23	85+-17	0.01	0.35	1.18

PC aa C38:5	HMDB0007990<br>HMDB0007989<br>HMDB0008050<br>HMDB0008114	51+-11	48+-11	0.43	0.70	1.05
PC aa C38:6	HMDB0008725<br>HMDB0008116<br>HMDB0008434<br>HMDB0007991<br>HMDB0008147<br>HMDB0008083<br>HMDB0008499	61+-15	59+-16	0.75	0.90	1.03
PC aa C40:2	HMDB0008308<br>HMDB0008276	0.45+-0.08	0.42+-0.11	0.34	0.70	1.06
PC aa C40:3	HMDB0008278<br>HMDB0008277<br>HMDB0008086<br>HMDB0008119	0.77+-0.13	0.76+-0.12	0.71	0.90	1.02
PC aa C40:4	HMDB0008279<br>HMDB0008628<br>HMDB0008054	4+-1	3.6+-0.8	0.08	0.56	1.13
PC aa C40:5	HMDB0008056<br>HMDB0008055<br>HMDB0008120	9.8+-2.1	8.6+-2	0.05	0.51	1.13
PC aa C40:6	HMDB08057<br>HMDB0008089	20+-5	18+-4	0.17	0.57	1.11
PC aa C42:0	HMDB0008537<br>HMDB0008760<br>HMDB0008282<br>HMDB0008058	0.46+-0.1	0.48+-0.09	0.34	0.70	0.95
PC aa C42:1	HMDB0008762<br>HMDB0008124<br>HMDB0008283<br>HMDB0008538<br>HMDB0008059	0.26+-0.09	0.28+-0.08	0.45	0.71	0.93
PC aa C42:2	HMDB0008157<br>HMDB0008795<br>HMDB0008125<br>HMDB0008763<br>HMDB0008092<br>HMDB0008316<br>HMDB0008794<br>HMDB0008602<br>HMDB0008284<br>HMDB0008348<br>HMDB0008539<br>HMDB0011330<br>HMDB0008570	0.25+-0.06	0.24+-0.07	0.70	0.90	1.03
PC aa C42:4	HMDB0008191	0.26+-0.09	0.23+-0.08	0.25	0.64	1.13
PC aa C42:5	HMDB08287	0.33+-0.09	0.35+-0.11	0.54	0.79	0.95
PC aa C42:6	HMDB0008288	0.42+-0.09	0.41+-0.14	0.61	0.85	1.04
PC ae C30:0	HMDB0013341	0.55+-0.21	0.61+-0.11	0.09	0.56	0.90
PC ae C30:1	HMDB0013402	0.72+-0.16	0.78+-0.11	0.16	0.57	0.93

PC ae C32:1	HMDB0013404<br>HMDB0007896<br>HMDB0007994	2.6+-0.6	2.7+-0.5	0.33	0.70	0.94
PC ae C32:2	HMDB0013411	1.2+-0.3	1.28+-0.23	0.58	0.82	0.96
PC ae C34:0	HMDB0013405	1.9+-0.6	2+-0.5	0.38	0.70	0.93
PC ae C34:1	HMDB0013426	9.2+-2.3	10.2+-1.9	0.13	0.56	0.91
PC ae C34:2	HMDB0011151	10.6+-2.4	11.2+-2.2	0.37	0.70	0.95
PC ae C34:3	HMDB0013413	8.1+-1.8	8.4+-1.9	0.57	0.82	0.96
PC ae C36:0	HMDB13406<br>HMDB0013417	1.2+-0.3	1.34+-0.25	0.21	0.62	0.93
PC ae C36:1	HMDB0013427<br>HMDB0013414	7.6+-1.8	8.6+-1.6	0.06	0.51	0.89
PC ae C36:2	HMDB0013418<br>HMDB0013428<br>HMDB0011243	13+-3	14+-3	0.35	0.70	0.94
PC ae C36:3	HMDB0013429<br>HMDB0013425	8.1+-1.9	8.1+-1.5	0.96	0.97	1.00
PC ae C36:4	HMDB0013435<br>HMDB0013407	15+-4	13+-4	0.14	0.56	1.14
PC ae C36:5	HMDB0013415<br>HMDB0011220	31+-7	30+-5	0.74	0.90	1.02
PC ae C38:0	HMDB0013408<br>HMDB0013419	1.9+-0.5	2.1+-0.5	0.18	0.57	0.90
PC ae C38:1	HMDB0013419<br>HMDB0013408	1.4+-0.3	1.5+-0.3	0.03	0.39	0.88
PC ae C38:2	HMDB0013416<br>HMDB0013430	2.3+-0.5	2.6+-0.5	0.07	0.56	0.90
PC ae C38:3	HMDB0013436<br>HMDB0013431	4+-0.9	4.3+-0.7	0.31	0.70	0.94
PC ae C38:4	HMDB0013420	13+-3	12.2+-2.1	0.53	0.79	1.04
PC ae C38:5	HMDB11253<br>HMDB0013432	16+-3	15+-3	0.06	0.51	1.12
PC ae C38:6	HMDB0013409	6.4+-1.3	5.9+-1.7	0.37	0.70	1.07
PC ae C40:1	HMDB0013433	1.09+-0.21	1.2+-0.3	0.13	0.56	0.91
PC ae C40:2	HMDB0013437	1.4+-0.3	1.6+-0.3	0.14	0.56	0.91
PC ae C40:3	HMDB0013445<br>HMDB0013446	1.03+-0.17	1.11+-0.16	0.07	0.56	0.92
PC ae C40:4	HMDB0013442	2.1+-0.4	2.2+-0.4	0.71	0.90	0.98
PC ae C40:5	HMDB0013444	3+-0.4	3+-0.5	0.81	0.95	0.99
PC ae C40:6	HMDB0013422	3.7+-0.8	3.7+-0.8	0.93	0.96	0.99
PC ae C42:1	HMDB0013434<br>HMDB0013447	0.34+-0.07	0.32+-0.06	0.27	0.67	1.07
PC ae C42:2	HMDB0013438	0.53+-0.13	0.57+-0.09	0.12	0.56	0.91
**PC ae C42:3**	**HMDB0013458<br>HMDB0013459**	**0.7+-0.1**	**0.82+-0.12**	**0.00**	**0.03**	**0.86**
PC ae C44:3	HMDB0013449<br>HMDB0013452	0.15+-0.06	0.15+-0.06	0.86	0.95	0.98
PC ae C44:4	HMDB0013455<br>HMDB0013453<br>HMDB0013460	0.46+-0.1	0.49+-0.11	0.32	0.70	0.94
PC ae C44:5	HMDB0013456	1.6+-0.4	1.7+-0.4	0.17	0.57	0.91
PC ae C44:6	HMDB0013450<br>HMDB0013457	0.99+-0.17	1+-0.24	0.80	0.95	0.98

Phenylalanine	HMDB00159	66+-8	70+-22	0.34	0.70	0.93
Proline	HMDB00162 HMDB03411	230+-60	200+-40	0.11	0.56	1.12
Putrescine	HMDB01414	0.16+-0.07	0.19+-0.06	0.14	0.56	0.85
Serine	HMDB00187 HMDB03406	117+-22	121+-20	0.45	0.71	0.96
Serotonin	HMDB00259	0.8+-0.4	0.9+-0.4	0.39	0.70	0.89
SM (OH) C14:1	HMDB0013462	5.8+-2	6.5+-1.6	0.23	0.62	0.90
SM (OH) C16:1	HMDB0013463	4.2+-1.2	4.2+-0.9	0.81	0.95	0.98
SM (OH) C22:1	HMDB0013466	17+-4	19+-4	0.30	0.70	0.94
SM (OH) C22:2	HMDB0013467	14+-3	15+-3	0.19	0.57	0.92
SM (OH) C24:1	HMDB0013469	1.8+-0.4	1.9+-0.5	0.62	0.85	0.96
SM C16:0	HMDB0061712 HMDB0010169	146+-22	140+-20	0.35	0.70	1.04
SM C16:1	HMDB0029216	21+-4	20+-3	0.35	0.70	1.05
SM C18:0	HMDB01348	29+-7	28+-6	0.67	0.88	1.03
SM C18:1	HMDB0012100 HMDB0012101	14+-4	13+-3	0.44	0.70	1.06
SM C20:2		0.5+-0.18	0.5+-0.16	0.94	0.96	0.99
SM C22:3		2.7+-1.1	2.6+-1.1	0.85	0.95	1.02
SM C24:0	HMDB11697	37+-6	36+-6	0.43	0.70	1.04
SM C24:1	HMDB12107	70+-11	65+-11	0.14	0.56	1.08
SM C26:0	HMDB0011698	0.38+-0.15	0.41+-0.15	0.54	0.79	0.94
SM C26:1	HMDB0013461	0.44+-0.21	0.41+-0.15	0.63	0.85	1.06
**Taurine**	**HMDB00251**	**61+-12**	**83+-20**	**0.00**	**0.01**	**0.74**
Threonine	HMDB04041 HMDB00167	121+-24	130+-30	0.41	0.70	0.95
Tryptophane	HMDB00929 HMDB0013609	64+-13	70+-10	0.13	0.56	0.93
Tyrosine	HMDB00158	69+-16	68+-12	0.78	0.93	1.02
Valine	HMDB00883	240+-50	270+-50	0.03	0.39	0.89
BCAA		416+-87	460+-85			
AAA		277+-30	295+-33			
Fischer ratio		1.5+-0.25	1.57+-0.27			
ADMA/Arg		0.0046+-0.001	0.004+-0.001			
Cit/Arg		0.26+-0.07	0.27+-0.05			
Cit/Orn		0.94+-0.49	1.3+-2.3			
Orn/Arg		0.33+-0.14	0.39+-0.21			

Table S1 b. Values of amino acids, of biogenic amines and their derivatives, and of selected sums and ratios – patients with preserved systolic ventricular function (group AF).

Metabolite	HMBD ID	Patients	Controls	p	q	FC
ADMA	HMDB01539	0.54+-0.12	0.48+-0.13	0.12	0.73	1.13
Alanine	HMDB00161 HMDB01310	400+-90	390+-80	0.59	0.84	1.03
Arginine	HMDB00517 HMDB03416	119+-14	119+-20	0.87	0.96	0.99
Asparagine	HMDB00168 HMDB003378	48+-10	48+-9	0.94	0.98	1.00
Aspartic acid	HMDB00191 HMDB06483	18.8+-2.4	20+-4	0.36	0.83	0.94
C0	HMDB00062	36+-7	33+-5	0.10	0.73	1.10
C14:1	HMDB0002014	0.14+-0.05	0.13+-0.03	0.71	0.90	1.03
C18:1	HMDB0006351 HMDB0005065 HMDB06464 HMDB0013338	0.15+-0.05	0.14+-0.03	0.57	0.84	1.05
C18:2	HMDB0006469 HMDB0006461	0.053+-0.02	0.049+-0.018	0.50	0.84	1.08
C2	HMDB00201	7+-3	6.5+-1.7	0.71	0.90	1.04
Citrulline	HMDB00904	30+-9	32+-7	0.57	0.84	0.95
Creatinine	HMDB0000562	72+-16	82+-14	0.04	0.50	0.88
Glutamine	HMDB00641 HMDB03423	740+-80	740+-110	0.88	0.96	1.01
Glutamic acid	HMDB00148 HMDB03339	47+-23	33+-20	0.04	0.50	1.42
Glycine	HMDB00123	270+-60	310+-80	0.06	0.59	0.87
H1		3800+-1300	3700+-600	0.79	0.95	1.02
Histidine	**HMDB00177**	**78+-10**	**87+-7**	**0.00**	**0.03**	**0.89**
Isoleucine	HMDB00172 HMDB0000557	71+-14	73+-13	0.59	0.84	0.97
Kynurenine	HMDB00684	4.5+-0.7	4.8+-0.9	0.16	0.73	0.93
Leucine	HMDB00687 HMDB0013773	100+-30	110+-30	0.31	0.81	0.92
Lysine	HMDB00182 HMDB03405	360+-30	320+-50	0.01	0.16	1.13
lysoPC a C16:0	HMDB10382 HMDB0061702	83+-13	83+-17	0.95	0.98	1.00
lysoPC a C16:1	HMDB0010383	3+-1.1	3.2+-0.8	0.53	0.84	0.94
lysoPC a C17:0	HMDB12108	7+-1	7.4+-1.6	0.27	0.77	0.94
lysoPC a C18:0	HMDB10384 HMDB0011128	28+-6	28+-5	0.88	0.96	1.01
lysoPC a C18:1	HMDB02815 HMDB0010385 HMDB0061701	23+-6	26+-8	0.21	0.75	0.90
lysoPC a C18:2	HMDB10386 HMDB0061700	33+-9	37+-15	0.29	0.81	0.89
lysoPC a C20:3	HMDB10394 HMDB0010393	3+-0.9	3+-0.8	0.98	0.98	1.00

lysoPC a C20:4	HMDB10395<br>HMDB0010396	8.3+-2	8+-3	0.50	0.84	1.06
lysoPC a C28:0	HMDB29206	0.5+-0.3	0.6+-0.3	0.13	0.73	0.77
lysoPC a C28:1	HMDB0029221	0.6+-0.4	0.8+-0.4	0.04	0.50	0.68
Methionine	HMDB00696	29+-4	30+-4	0.49	0.84	0.97
Ornithine	HMDB00214<br>HMDB03374	41+-16	46+-23	0.37	0.83	0.88
PC aa C28:1	HMDB0007867<br>HMDB0007899	3+-0.8	3.3+-0.7	0.15	0.73	0.90
PC aa C30:0	HMDB0007934<br>HMDB0007869<br>HMDB0007965	12+-3	12.1+-2.2	0.82	0.96	1.02
PC aa C30:2	HMDB0007999	1.2+-0.23	1.1+-0.3	0.20	0.75	1.09
PC aa C32:0	HMDB00564<br>HMDB0007871<br>HMDB0008031	14+-4	14+-3	0.44	0.83	1.06
PC aa C32:1	HMDB0007872<br>HMDB0007873<br>HMDB0008097<br>HMDB0007969	17+-11	17+-7	0.98	0.98	1.00
PC aa C32:2	HMDB0008002<br>HMDB0007874	4.6+-1.9	5+-1.7	0.50	0.84	0.93
PC aa C32:3	HMDB0007876	0.69+-0.16	0.74+-0.13	0.27	0.77	0.93
PC aa C34:1	HMDB0007971<br>HMDB08100<br>HMDB0008035<br>HMDB0007972<br>HMDB0008003	240+-60	250+-50	0.67	0.89	0.97
PC aa C34:2	HMDB07973<br>HMDB0008101<br>HMDB0008133<br>HMDB0008005<br>HMDB0008004	390+-50	390+-70	0.97	0.98	1.00
PC aa C34:3	HMDB08006<br>HMDB0007974<br>HMDB0007975	14+-4	16+-6	0.20	0.75	0.87
PC aa C34:4	HMDB0007883<br>HMDB0007976	1.9+-0.9	2+-0.7	0.83	0.96	0.97
PC aa C36:0	HMDB0008265<br>HMDB0008036<br>HMDB0008525<br>HMDB0007886	5.9+-1.3	6+-1.1	0.67	0.89	0.97
PC aa C36:1	HMDB08038<br>HMDB0008069<br>HMDB0007978<br>HMDB0008102	62+-14	65+-13	0.42	0.83	0.95
PC aa C36:2	HMDB08039<br>HMDB0008070<br>HMDB08135<br>HMDB0007979	230+-40	230+-40	0.87	0.96	1.01

PC aa C36:3	HMDB08105<br>HMDB0007980<br>HMDB0007981<br>HMDB0008040	130+-30	140+-30	0.78	0.95	0.98
PC aa C36:4	HMDB07982<br>HMDB0008107<br>HMDB0008138<br>HMDB0008234<br>HMDB0008429<br>HMDB0008170<br>HMDB0008138<br>HMDB0008042<br>HMDB0008203<br>HMDB0008106	170+-40	160+-30	0.13	0.73	1.11
PC aa C36:5	HMDB07984<br>HMDB0008015	20+-14	21+-12	0.79	0.95	0.95
PC aa C36:6	HMDB0008206<br>HMDB0007892	0.9+-0.4	0.9+-0.4	0.52	0.84	0.92
PC aa C38:0	HMDB0007893<br>HMDB0008528<br>HMDB0008043<br>HMDB0008755<br>HMDB0008267<br>HMDB0007985	3.4+-0.6	3.2+-0.7	0.53	0.84	1.04
PC aa C38:1	HMDB0007894<br>HMDB0008269<br>HMDB0007986<br>HMDB0008268<br>HMDB0008109<br>HMDB0008044	0.14+-0.18	0.22+-0.24	0.22	0.75	0.64
PC aa C38:3	HMDB0008020<br>HMDB0008046<br>HMDB0008047	50+-12	47+-11	0.42	0.83	1.06
PC aa C38:4	HMDB0008048<br>HMDB0008112<br>HMDB0008113<br>HMDB0007988	101+-24	85+-17	0.02	0.46	1.18
PC aa C38:5	HMDB0007990<br>HMDB0007989<br>HMDB0008050<br>HMDB0008114	51+-11	49+-11	0.58	0.84	1.04
PC aa C38:6	HMDB0008725<br>HMDB0008116<br>HMDB0008434<br>HMDB0007991<br>HMDB0008147<br>HMDB0008083<br>HMDB0008499	60+-16	59+-17	0.81	0.96	1.02
PC aa C40:2	HMDB0008308<br>HMDB0008276	0.45+-0.08	0.42+-0.11	0.44	0.83	1.06
PC aa C40:3	HMDB0008278<br>HMDB0008277<br>HMDB0008086<br>HMDB0008119	0.77+-0.14	0.77+-0.13	0.92	0.98	1.01

PC aa C40:4	HMDB0008279<br>HMDB0008628<br>HMDB0008054	4+-1	3.6+-0.8	0.15	0.73	1.11
PC aa C40:5	HMDB0008056<br>HMDB0008055<br>HMDB0008120	9.8+-2.2	8.8+-2	0.13	0.73	1.11
PC aa C40:6	HMDB08057<br>HMDB0008089	20+-5	18+-4	0.23	0.75	1.10
PC aa C42:0	HMDB0008537<br>HMDB0008760<br>HMDB0008282<br>HMDB0008058	0.46+-0.1	0.48+-0.09	0.48	0.84	0.96
PC aa C42:1	HMDB0008762<br>HMDB0008124<br>HMDB0008283<br>HMDB0008538<br>HMDB0008059	0.26+-0.09	0.28+-0.08	0.44	0.83	0.93
PC aa C42:2	HMDB0008157<br>HMDB0008795<br>HMDB0008125<br>HMDB0008763<br>HMDB0008092<br>HMDB0008316<br>HMDB0008794<br>HMDB0008602<br>HMDB0008284<br>HMDB0008348<br>HMDB0008539<br>HMDB0011330<br>HMDB0008570	0.25+-0.06	0.24+-0.07	0.58	0.84	1.05
PC aa C42:4	HMDB0008191	0.26+-0.09	0.23+-0.08	0.21	0.75	1.15
PC aa C42:5	HMDB08287	0.33+-0.09	0.36+-0.11	0.43	0.83	0.93
PC aa C42:6	HMDB0008288	0.42+-0.09	0.41+-0.14	0.67	0.89	1.04
PC ae C30:0	HMDB0013341	0.55+-0.21	0.61+-0.12	0.16	0.73	0.90
PC ae C30:1	HMDB0013402	0.72+-0.16	0.79+-0.11	0.11	0.73	0.91
PC ae C32:1	HMDB0013404<br>HMDB0007896<br>HMDB0007994	2.6+-0.6	2.7+-0.5	0.33	0.81	0.94
PC ae C32:2	HMDB0013411	1.2+-0.3	1.29+-0.24	0.49	0.84	0.95
PC ae C34:0	HMDB0013405	1.9+-0.6	2.1+-0.5	0.40	0.83	0.93
PC ae C34:1	HMDB0013426	9.3+-2.4	10.2+-1.9	0.17	0.73	0.91
PC ae C34:2	HMDB0011151	11+-3	11.2+-2.3	0.33	0.81	0.94
PC ae C34:3	HMDB0013413	8+-1.9	8.5+-2	0.45	0.84	0.95
PC ae C36:0	HMDB13406<br>HMDB0013417	1.2+-0.3	1.4+-0.3	0.23	0.75	0.93
PC ae C36:1	HMDB0013427<br>HMDB0013414	7.7+-1.8	8.7+-1.7	0.06	0.63	0.88
PC ae C36:2	HMDB0013418<br>HMDB0013428<br>HMDB0011243	13+-3	14+-3	0.39	0.83	0.95
PC ae C36:3	HMDB0013429<br>HMDB0013425	7.9+-1.9	8.1+-1.6	0.78	0.95	0.98
PC ae C36:4	HMDB0013435<br>HMDB0013407	15+-4	13+-4	0.27	0.77	1.11

PC ae C36:5	HMDB0013415<br>HMDB0011220	30+-7	30+-5	0.84	0.96	1.01
PC ae C38:0	HMDB0013408<br>HMDB0013419	1.9+-0.6	2.1+-0.6	0.21	0.75	0.90
PC ae C38:1	HMDB0013419<br>HMDB0013408	1.4+-0.3	1.6+-0.3	0.03	0.50	0.88
PC ae C38:2	HMDB0013416<br>HMDB0013430	2.3+-0.5	2.6+-0.5	0.12	0.73	0.91
PC ae C38:3	HMDB0013436<br>HMDB0013431	4+-0.9	4.3+-0.7	0.33	0.81	0.94
PC ae C38:4	HMDB0013420	13+-3	12.1+-2.1	0.56	0.84	1.04
PC ae C38:5	HMDB11253<br>HMDB0013432	16+-3	14+-3	0.10	0.73	1.11
PC ae C38:6	HMDB0013409	6.3+-1.4	6+-1.8	0.57	0.84	1.05
PC ae C40:1	HMDB0013433	1.09+-0.22	1.2+-0.3	0.17	0.73	0.91
PC ae C40:2	HMDB0013437	1.4+-0.3	1.6+-0.3	0.12	0.73	0.90
PC ae C40:3	HMDB0013445<br>HMDB0013446	1.03+-0.17	1.12+-0.17	0.09	0.73	0.92
PC ae C40:4	HMDB0013442	2.1+-0.4	2.1+-0.4	0.79	0.95	0.99
PC ae C40:5	HMDB0013444	3+-0.4	3+-0.5	0.89	0.96	0.99
PC ae C40:6	HMDB0013422	3.7+-0.8	3.7+-0.8	0.85	0.96	0.99
PC ae C42:1	HMDB0013434<br>HMDB0013447	0.33+-0.07	0.32+-0.06	0.50	0.84	1.04
PC ae C42:2	HMDB0013438	0.52+-0.13	0.57+-0.09	0.19	0.75	0.92
PC ae C42:3	**HMDB0013458<br>HMDB0013459**	**0.69+-0.1**	**0.82+-0.12**	**0.00**	**0.02**	**0.84**
PC ae C44:3	HMDB0013449<br>HMDB0013452	0.15+-0.06	0.15+-0.06	0.97	0.98	1.00
PC ae C44:4	HMDB0013455<br>HMDB0013453<br>HMDB0013460	0.46+-0.1	0.47+-0.09	0.66	0.89	0.97
PC ae C44:5	HMDB0013456	1.6+-0.4	1.7+-0.4	0.35	0.82	0.93
PC ae C44:6	HMDB0013450<br>HMDB0013457	0.99+-0.18	0.99+-0.24	0.95	0.98	1.00
Phenylalanine	HMDB00159	66+-8	70+-23	0.43	0.83	0.94
Proline	HMDB00162<br>HMDB03411	230+-50	200+-40	0.03	0.50	1.17
Putrescine	HMDB01414	0.17+-0.07	0.19+-0.06	0.32	0.81	0.89
Serine	HMDB00187<br>HMDB03406	118+-22	119+-18	0.87	0.96	0.99
Serotonin	HMDB00259	0.8+-0.4	0.8+-0.3	0.68	0.89	0.94
SM (OH) C14:1	HMDB0013462	5.9+-2.1	6.6+-1.6	0.25	0.76	0.90
SM (OH) C16:1	HMDB0013463	4.2+-1.2	4.3+-0.9	0.68	0.89	0.97
SM (OH) C22:1	HMDB0013466	17+-4	19+-4	0.31	0.81	0.93
SM (OH) C22:2	HMDB0013467	14+-3	16+-3	0.17	0.73	0.92
SM (OH) C24:1	HMDB0013469	1.8+-0.4	1.9+-0.5	0.70	0.90	0.97
SM C16:0	HMDB0061712<br>HMDB0010169	146+-23	140+-20	0.41	0.83	1.04
SM C16:1	HMDB0029216	21+-4	20+-3	0.34	0.82	1.05
SM C18:0	HMDB01348	28+-7	28+-6	0.92	0.98	1.01

SM C18:1	HMDB0012100<br>HMDB0012101	14+-3	13+-3	0.64	0.89	1.04
SM C20:2		0.49+-0.19	0.51+-0.16	0.73	0.92	0.96
SM C22:3		2.7+-1.2	2.6+-1.2	0.95	0.98	1.01
SM C24:0	HMDB11697	37+-6	36+-7	0.52	0.84	1.03
SM C24:1	HMDB12107	69+-12	65+-12	0.27	0.77	1.06
SM C26:0	HMDB0011698	0.39+-0.16	0.42+-0.15	0.49	0.84	0.92
SM C26:1	HMDB0013461	0.44+-0.22	0.41+-0.16	0.58	0.84	1.08
**Taurine**	**HMDB00251**	**61+-11**	**84+-21**	**0.00**	**0.01**	**0.72**
Threonine	HMDB04041<br>HMDB00167	121+-25	120+-30	0.60	0.84	0.97
Tryptophane	HMDB00929<br>HMDB0013609	65+-13	69+-10	0.24	0.75	0.94
Tyrosine	HMDB00158	71+-16	67+-12	0.40	0.83	1.05
Valine	HMDB00883	240+-48	270+-40	0.05	0.59	0.90
BCAA		416+-90	455+-81			
AAA		279+-30	294+-34			
Fischer ratio		1.49+-0.25	1.56+-0.26			
ADMA/Arg		0.0046+-0.001	0.004+-0.001			
Cit/Arg		0.26+-0.08	0.27+-0.05			
Cit/Orn		0.85+-0.38	1.34+-2.3			
Orn/Arg		0.35+-0.13	0.39+-0.21			

Table S1 c. Values of amino acids, of biogenic amines and their derivatives, and of selected sums and ratios – patients with preserved systolic function and without major valve regurgitation (group AFV).

Metabolite	HMBD ID	Patients	Controls	p	q	FC
ADMA	HMDB01539	0.53+-0.12	0.48+-0.1	0.28	0.84	1.10
Alanine	HMDB00161 HMDB01310	430+-70	380+-80	0.14	0.84	1.11
Arginine	HMDB00517 HMDB03416	123+-13	121+-20	0.71	0.92	1.02
Asparagine	HMDB00168 HMDB003378	50+-12	51+-7	0.69	0.92	0.97
Aspartic acid	HMDB00191 HMDB06483	18.9+-2.4	20+-4	0.43	0.85	0.95
C0	HMDB00062	36+-9	33+-4	0.31	0.84	1.08
C14:1	HMDB0002014	0.13+-0.04	0.14+-0.03	0.47	0.85	0.93
C18:1	HMDB0006351 HMDB0005065 HMDB06464 HMDB0013338	0.14+-0.05	0.15+-0.04	0.65	0.92	0.95
C18:2	HMDB0006469 HMDB0006461	0.049+-0.022	0.052+-0.02	0.79	0.92	0.96
C2	HMDB00201	6+-3	6.6+-1.6	0.57	0.91	0.93
Citrulline	HMDB00904	31+-9	33+-7	0.57	0.91	0.94
Creatinine	HMDB0000562	76+-19	83+-17	0.27	0.84	0.91
Glutamine	HMDB00641 HMDB03423	750+-80	730+-110	0.58	0.91	1.03
Glutamic acid	HMDB00148 HMDB03339	45+-23	35+-24	0.24	0.84	1.30
Glycine	HMDB00123	270+-50	290+-50	0.19	0.84	0.91
H1		3900+-1600	3700+-400	0.61	0.92	1.06
**Histidine**	**HMDB00177**	**81+-10**	**87+-9**	**0.07**	**0.84**	**0.92**
Isoleucine	HMDB00172 HMDB0000557	76+-15	74+-12	0.66	0.92	1.03
Kynurenine	HMDB00684	4.4+-0.8	4.6+-0.8	0.45	0.85	0.95
Leucine	HMDB00687 HMDB0013773	120+-30	110+-30	0.95	0.98	1.01
Lysine	HMDB00182 HMDB03405	370+-40	320+-60	0.01	0.29	1.16
lysoPC a C16:0	HMDB10382 HMDB0061702	85+-14	80+-11	0.35	0.84	1.06
lysoPC a C16:1	HMDB0010383	3.2+-1	3+-0.5	0.61	0.92	1.05
lysoPC a C17:0	HMDB12108	7.1+-1	7.3+-1.1	0.66	0.92	0.98
lysoPC a C18:0	HMDB10384 HMDB0011128	28+-6	28+-4	0.83	0.92	1.02
lysoPC a C18:1	HMDB02815 HMDB0010385 HMDB0061701	24+-6	26+-6	0.41	0.84	0.93
lysoPC a C18:2	HMDB10386 HMDB0061700	35+-10	38+-15	0.56	0.91	0.92
lysoPC a C20:3	HMDB10394 HMDB0010393	3.1+-1	3+-0.9	0.81	0.92	1.03

lysoPC a C20:4	HMDB10395 HMDB0010396	9+-2	8+-3	0.38	0.84	1.10
lysoPC a C28:0	HMDB29206	0.5+-0.3	0.6+-0.3	0.33	0.84	0.80
lysoPC a C28:1	HMDB0029221	0.6+-0.4	0.7+-0.5	0.40	0.84	0.82
Methionine	HMDB00696	30+-4	31+-4	0.58	0.91	0.97
Ornithine	HMDB00214 HMDB03374	42+-16	51+-25	0.23	0.84	0.81
PC aa C28:1	HMDB0007867 HMDB0007899	3+-0.8	3.1+-0.6	0.67	0.92	0.96
PC aa C30:0	HMDB0007934 HMDB0007869 HMDB0007965	12+-3	11.3+-1.6	0.36	0.84	1.08
PC aa C30:2	HMDB0007999	1.21+-0.24	1+-0.3	0.08	0.84	1.17
PC aa C32:0	HMDB00564 HMDB0007871 HMDB0008031	14+-4	12.8+-2.4	0.20	0.84	1.14
PC aa C32:1	HMDB0007872 HMDB0007873 HMDB0008097 HMDB0007969	17+-12	14+-6	0.41	0.84	1.21
PC aa C32:2	HMDB0008002 HMDB0007874	4.8+-2.1	4.3+-1.6	0.51	0.89	1.11
PC aa C32:3	HMDB0007876	0.69+-0.18	0.71+-0.13	0.74	0.92	0.97
PC aa C34:1	HMDB0007971 HMDB08100 HMDB0008035 HMDB0007972 HMDB0008003	240+-60	230+-40	0.48	0.85	1.06
PC aa C34:2	HMDB07973 HMDB0008101 HMDB0008133 HMDB0008005 HMDB0008004	400+-50	370+-60	0.10	0.84	1.10
PC aa C34:3	HMDB08006 HMDB0007974 HMDB0007975	14+-5	15+-7	0.74	0.92	0.95
PC aa C34:4	HMDB0007883 HMDB0007976	2+-1	1.7+-0.6	0.34	0.84	1.18
PC aa C36:0	HMDB0008265 HMDB0008036 HMDB0008525 HMDB0007886	5.9+-1.3	5.8+-1	0.74	0.92	1.02
PC aa C36:1	HMDB08038 HMDB0008069 HMDB0007978 HMDB0008102	63+-14	62+-13	0.93	0.97	1.01
PC aa C36:2	HMDB08039 HMDB0008070 HMDB08135 HMDB0007979	240+-40	220+-40	0.25	0.84	1.08
PC aa C36:3	HMDB08105 HMDB0007980 HMDB0007981 HMDB0008040	140+-30	124+-23	0.20	0.84	1.10

PC aa C36:4	HMDB07982<br>HMDB0008107<br>HMDB0008138<br>HMDB0008234<br>HMDB0008429<br>HMDB0008170<br>HMDB0008138<br>HMDB0008042<br>HMDB0008203<br>HMDB0008106	190+-40	150+-30	0.01	0.29	1.24
PC aa C36:5	HMDB07984<br>HMDB0008015	19+-11	18+-8	0.71	0.92	1.08
PC aa C36:6	HMDB0008206<br>HMDB0007892	0.9+-0.4	0.8+-0.3	0.57	0.91	1.09
PC aa C38:0	HMDB0007893<br>HMDB0008528<br>HMDB0008043<br>HMDB0008755<br>HMDB0008267<br>HMDB0007985	3.4+-0.7	3.2+-0.7	0.39	0.84	1.07
PC aa C38:1	HMDB0007894<br>HMDB0008269<br>HMDB0007986<br>HMDB0008268<br>HMDB0008109<br>HMDB0008044	0.15+-0.2	0.2+-0.3	0.42	0.85	0.67
PC aa C38:3	HMDB0008020<br>HMDB0008046<br>HMDB0008047	50+-12	44+-10	0.16	0.84	1.14
PC aa C38:4	HMDB0008048<br>HMDB0008112<br>HMDB0008113<br>HMDB0007988	106+-24	84+-17	0.01	0.29	1.26
PC aa C38:5	HMDB0007990<br>HMDB0007989<br>HMDB0008050<br>HMDB0008114	51+-10	47+-10	0.25	0.84	1.09
PC aa C38:6	HMDB0008725<br>HMDB0008116<br>HMDB0008434<br>HMDB0007991<br>HMDB0008147<br>HMDB0008083<br>HMDB0008499	60+-15	55+-16	0.45	0.85	1.08
PC aa C40:2	HMDB0008308<br>HMDB0008276	0.45+-0.07	0.4+-0.12	0.21	0.84	1.12
PC aa C40:3	HMDB0008278<br>HMDB0008277<br>HMDB0008086<br>HMDB0008119	0.76+-0.11	0.73+-0.1	0.41	0.84	1.05
PC aa C40:4	HMDB0008279<br>HMDB0008628<br>HMDB0008054	4+-0.9	3.5+-0.8	0.12	0.84	1.15
PC aa C40:5	HMDB0008056<br>HMDB0008055<br>HMDB0008120	9.5+-1.7	8.6+-2	0.18	0.84	1.11
PC aa C40:6	HMDB08057<br>HMDB0008089	19+-5	17+-4	0.21	0.84	1.13

PC aa C42:0	HMDB0008537 HMDB0008760 HMDB0008282 HMDB0008058	0.45+-0.09	0.49+-0.09	0.28	0.84	0.92
PC aa C42:1	HMDB0008762 HMDB0008124 HMDB0008283 HMDB0008538 HMDB0008059	0.27+-0.08	0.28+-0.09	0.73	0.92	0.96
PC aa C42:2	HMDB0008157 HMDB0008795 HMDB0008125 HMDB0008763 HMDB0008092 HMDB0008316 HMDB0008794 HMDB0008602 HMDB0008284 HMDB0008348 HMDB0008539 HMDB0011330 HMDB0008570	0.24+-0.05	0.24+-0.05	0.79	0.92	0.98
PC aa C42:4	HMDB0008191	0.26+-0.09	0.22+-0.07	0.28	0.84	1.16
PC aa C42:5	HMDB08287	0.33+-0.11	0.34+-0.11	0.85	0.93	0.98
PC aa C42:6	HMDB0008288	0.41+-0.09	0.37+-0.14	0.35	0.84	1.12
PC ae C30:0	HMDB0013341	0.52+-0.24	0.59+-0.11	0.32	0.84	0.88
PC ae C30:1	HMDB0013402	0.71+-0.18	0.77+-0.12	0.36	0.84	0.92
PC ae C32:1	HMDB0013404 HMDB0007896 HMDB0007994	2.7+-0.7	2.7+-0.5	0.83	0.92	0.98
PC ae C32:2	HMDB0013411	1.2+-0.4	1.23+-0.22	0.97	0.98	1.00
PC ae C34:0	HMDB0013405	1.9+-0.7	2+-0.4	0.76	0.92	0.97
PC ae C34:1	HMDB0013426	9+-3	9.9+-1.9	0.46	0.85	0.93
PC ae C34:2	HMDB0011151	11+-3	11.4+-2.4	0.53	0.89	0.94
PC ae C34:3	HMDB0013413	8.4+-2	8.5+-1.9	0.88	0.95	0.99
PC ae C36:0	HMDB13406 HMDB0013417	1.2+-0.3	1.3+-0.3	0.36	0.84	0.93
PC ae C36:1	HMDB0013427 HMDB0013414	7.5+-2	8.2+-1.4	0.30	0.84	0.92
PC ae C36:2	HMDB0013418 HMDB0013428 HMDB0011243	13+-3	13+-3	0.77	0.92	0.97
PC ae C36:3	HMDB0013429 HMDB0013425	8.3+-2.1	8.1+-1.7	0.79	0.92	1.02
PC ae C36:4	HMDB0013435 HMDB0013407	16+-5	14+-5	0.27	0.84	1.15
PC ae C36:5	HMDB0013415 HMDB0011220	31+-7	28+-4	0.18	0.84	1.11
PC ae C38:0	HMDB0013408 HMDB0013419	1.9+-0.6	2+-0.4	0.75	0.92	0.97
PC ae C38:1	HMDB0013419 HMDB0013408	1.4+-0.3	1.5+-0.3	0.38	0.84	0.93
PC ae C38:2	HMDB0013416 HMDB0013430	2.3+-0.6	2.4+-0.5	0.66	0.92	0.96
PC ae C38:3	HMDB0013436 HMDB0013431	4+-1	4+-0.6	0.93	0.97	0.99
PC ae C38:4	HMDB0013420	13+-3	12.2+-2.3	0.39	0.84	1.08

PC ae C38:5	HMDB11253<br>HMDB0013432	17+-4	15+-3	0.11	0.84	1.15
PC ae C38:6	HMDB0013409	6.5+-1.3	6+-2	0.40	0.84	1.09
PC ae C40:1	HMDB0013433	1.14+-0.24	1.12+-0.23	0.89	0.95	1.01
PC ae C40:2	HMDB0013437	1.4+-0.4	1.5+-0.3	0.36	0.84	0.92
PC ae C40:3	HMDB0013445<br>HMDB0013446	1.02+-0.19	1.1+-0.17	0.31	0.84	0.93
PC ae C40:4	HMDB0013442	2.2+-0.4	2.1+-0.4	0.68	0.92	1.03
PC ae C40:5	HMDB0013444	3+-0.5	3+-0.5	0.82	0.92	1.01
PC ae C40:6	HMDB0013422	3.7+-0.9	3.6+-0.8	0.93	0.97	1.01
PC ae C42:1	HMDB0013434<br>HMDB0013447	0.34+-0.07	0.31+-0.06	0.20	0.84	1.10
PC ae C42:2	HMDB0013438	0.55+-0.15	0.58+-0.08	0.52	0.89	0.95
**PC ae C42:3**	**HMDB0013458<br>HMDB0013459**	**0.71+-0.11**	**0.81+-0.11**	**0.01**	**0.29**	**0.87**
PC ae C44:3	HMDB0013449<br>HMDB0013452	0.16+-0.07	0.15+-0.08	0.77	0.92	1.06
PC ae C44:4	HMDB0013455<br>HMDB0013453<br>HMDB0013460	0.47+-0.11	0.47+-0.08	0.99	0.99	1.00
PC ae C44:5	HMDB0013456	1.7+-0.4	1.7+-0.4	0.62	0.92	0.96
PC ae C44:6	HMDB0013450<br>HMDB0013457	1.02+-0.19	1.01+-0.24	0.89	0.95	1.01
Phenylalanine	HMDB00159	69+-7	68+-7	0.72	0.92	1.01
Proline	HMDB00162<br>HMDB03411	250+-50	200+-40	0.01	0.29	1.25
Putrescine	HMDB01414	0.16+-0.06	0.19+-0.07	0.21	0.84	0.84
Serine	HMDB00187<br>HMDB03406	116+-22	123+-21	0.39	0.84	0.94
Serotonin	HMDB00259	0.8+-0.3	0.9+-0.3	0.27	0.84	0.86
SM (OH) C14:1	HMDB0013462	5.5+-2	6.3+-1.5	0.24	0.84	0.87
SM (OH) C16:1	HMDB0013463	4+-1.1	4.2+-0.9	0.46	0.85	0.93
SM (OH) C22:1	HMDB0013466	18+-4	18+-4	0.94	0.97	0.99
SM (OH) C22:2	HMDB0013467	14+-3	15+-2.1	0.48	0.85	0.94
SM (OH) C24:1	HMDB0013469	1.8+-0.5	1.8+-0.5	0.96	0.98	1.00
SM C16:0	HMDB0061712<br>HMDB0010169	147+-22	140+-18	0.34	0.84	1.05
SM C16:1	HMDB0029216	21+-4	19+-3	0.09	0.84	1.12
SM C18:0	HMDB01348	29+-5	27+-6	0.59	0.92	1.04
SM C18:1	HMDB0012100<br>HMDB0012101	13.6+-2.3	13+-3	0.41	0.84	1.07
SM C20:2		0.5+-0.19	0.48+-0.15	0.70	0.92	1.05
SM C22:3		2.6+-0.7	2.4+-0.8	0.48	0.85	1.09
SM C24:0	HMDB11697	38+-7	35+-6	0.18	0.84	1.10
SM C24:1	HMDB12107	70+-9	65+-11	0.14	0.84	1.09
SM C26:0	HMDB0011698	0.42+-0.19	0.43+-0.16	0.80	0.92	0.96
SM C26:1	HMDB0013461	0.45+-0.19	0.44+-0.15	0.87	0.95	1.03
**Taurine**	**HMDB00251**	**61+-12**	**88+-20**	**0.00**	**0.03**	**0.69**
Threonine	HMDB04041<br>HMDB00167	125+-24	130+-30	0.69	0.92	0.97

Tryptophane	HMDB00929 HMDB0013609	66+-10	73+-10	0.08	0.84	0.91
Tyrosine	HMDB00158	76+-16	70+-11	0.30	0.84	1.08
Valine	HMDB00883	260+-50	270+-50	0.70	0.92	0.97
BCAA		450+-92	454+-72			
AAA		291+-23	298+-25			
Fischer ratio		1.55+-0.28	1.52+-0.22			
ADMA/Arg		0.0043+-0.001	0.0041+-0.001			
Cit/Arg		0.25+-0.07	0.27+-0.05			
Cit/Orn		0.82+-0.37	0.88+-0.7			
Orn/Arg		0.33+-0.11	0.44+-0.23			

Table S2. Multivariate regression analysis.

Table S2 a. Multivariate regression analysis, clinical and imaging parameters as outcome variables. Correlation of variables which were different between patients and controls and the outcome variables 'group assignment to patients vs. controls', 'evidence of diastolic dysfunction', 'history of aortic root surgery' and aortic root dimensions. False discovery rate-adjusted p (=q) ≤ 0.05 was considered statistically significant. Group A, all patients; group AF, patients with preserved systolic ventricular function; group AFV, patients with preserved systolic ventricular function and without major valve regurgitation. β, beta coefficient in multivariate regression; NT-proBNP, N-terminal prohormone of brain natriuretic peptide R^2, R squared referring to the regression analysis of the group of the variables listed straight above (corresponding line and column), respectively.

**Groups**	**Independent variables**	**Outcome variable: patients vs. controls** β/R^2 (q)
**A Pat. + Ctrl.**	NT-proBNP	0.002 (0.0055)
	PCaeC42:3	-0.07 (0.3)
	Taurine	-0.009 (0.004)
	Histidine	-0.02 (0.004)
	Adjusted R^2 (q)	0.53 (<0.0001)
**AF Pat. + Ctrl.**	NT-proBNP	0.002 (0.016)
	PCaeC42:3	-0.06 (0.35)
	Taurine	-0.009 (0.003)
	Histidine	-0.02 (0.01)
	Adjusted R^2 (q)	0.51 (<0.0001)
**AFV Pat. + Ctrl.**	NT-proBNP	0.002 (0.05)
	PCaeC42:3	0.05 (0.59)
	Taurine	-0.012 (0.003)
	Histidine	-0.007 (0.3)
	Adjusted R^2 (q)	0.52 (0.0004)
**Groups**	**Independent variables**	**Outcome variable: evidence of diastolic dysfunction** β/r^2 (q)
**A Pat.**	NT-proBNP	0.0007 (0.59)
	PCaeC42:3	-0.08 (0.65)
	Taurine	0.004 (0.77)
	Histidine	0.002 (0.89)
	Adjusted R^2 (q)	-0.2 (0.97)
**AF Pat.**	NT-proBNP	0.0004 (0.76)
	PCaeC42:3	-0.03 (0.88)
	Taurine	-0.002 (0.89)
	Histidine	0.0004 (0.98)
	Adjusted R^2 (q)	-0.24 (0.99)
**AFV Pat.**	NT-proBNP	-0.0018 (0.27)
	PCaeC42:3	0.21 (0.39)
	Taurine	0.018 (0.36)
	Histidine	0.02 (0.33)
	Adjusted R^2 (q)	-1.5 (0.67)
**Groups**	**Independent variables**	**Outcome variable: history of aortic root surgery** β/r^2 (q)
**A Pat.**	NT-proBNP	0.0002 (0.15)
	PCaeC42:3	0.03 (0.85)
	Taurine	-0.005 (0.77)
	Histidine	0.008 (0.89)
	Adjusted R^2 (q)	-0.03 (0.52)
**AF Pat.**	NT-proBNP	0.001 (0.19)
	PCaeC42:3	0.09 (0.57)
	Taurine	-0.01 (0.34)
	Histidine	0.01 (0.40)
	Adjusted R^2 (q)	-0.005 (0.49)
**AFV Pat.**	NT-proBNP	0.002 (0.18)
	PCaeC42:3	0.12 (0.52)
	Taurine	-0.006 (0.69)
	Histidine	0.008 (0.61)
	Adjusted R^2 (q)	-0.02 (0.47)
**Groups**	**Independent variables**	**Outcome variable: aortic root dimension** β/r^2 (q)
**A Pat.**	NT-proBNP	0.008 (0.23)
	PCaeC42:3	-0.69 (0.34)
	Taurine	0.02 (0.7)
	Histidine	-0.07 (0.37)
	Adjusted R^2 (q)	-0.08 (0.56)
**AF Pat.**	NT-proBNP	0.01 (0.16)
	PCaeC42:3	-0.2 (0.8)

Groups	Independent variables	
	Taurine<br>Histidine	-0.005 (0.9)<br>- 0.02 (0.87)
	Adjusted R^2 (q)	-0.08 (0.57)
**AFV Pat.**	NT-proBNP<br>PCaeC42:3<br>Taurine<br>Histidine	-0.003 (0.93)<br>-0.58 (0.7)<br>-0.02 (0.85)<br>-0.0007 (0.99)
	Adjusted R^2 (q)	-1.15 (0.85)
**Groups**	**Independent variables**	**Outcome variable: ascending aorta dimension**<br>**β/r^2 (q)**
**A Pat.**	NT-proBNP<br>PCaeC42:3<br>Taurine<br>Histidine	0.01 (0.008)<br>0.1 (0.85)<br>-0.004 (0.93)<br>-0.05 (0.36)
	Adjusted R^2 (q)	-0.42 (0.048)
**AF Pat.**	NT-proBNP<br>PCaeC42:3<br>Taurine<br>Histidine	0.01 (0.003)<br>-0.72 (0.17)<br>-0.04 (0.29)<br>0.002 (0.97)
	Adjusted R^2 (q)	0.6 (0.01)
**AFV Pat.**	NT-proBNP<br>PCaeC42:3<br>Taurine<br>Histidine	0.008 (0.11)<br>0.73 (0.37)<br>-0.04 (0.51)<br>0.02 (0.78)
	Adjusted R^2 (q)	0.45 (0.19)

Table S2 b. Multivariate regression analysis, biochemical parameters as outcome variables. Correlation of the variables 'diastolic dysfunction', 'history of surgery', and aortic root dimensions and the outcome variables 'serum concentration of Histidine', 'serum concentration of Taurine', 'serum concentration of PCaeC42:3' and 'serum concentration of NT-proBNP'. False discovery rate-adjusted p (=q) ≤ 0.05 was considered statistically significant. Group A, all patients; group AF, patients with preserved systolic ventricular function; group AFV, patients with preserved systolic ventricular function and without major valve regurgitation. Asc., ascending; β, beta coefficient in multivariate regression; dim., dimension; hx, history; NT-proBNP, N-terminal prohormone of brain natriuretic peptide R^2, R squared referring to the regression analysis of the group of the variables listed straight above (corresponding line and column), respectively; z-sc., z-score.

**Group**	**Independent variables**	**Outcome variable: Histidine** **β/R^2 (q)**
**A Pat.**	Diastolic dysfunction	-1.7 (0.76)
	Hx of surgery	-2.8 (0.72)
	Aortic root dim. z-sc.	-1.4 (0.61)
	Asc. aorta dim. z-sc.	-0.3 (0.89)
	Adjusted R^2 (q)	0.13 (0.88)
**AF Pat.**	Diastolic dysfunction	-1.3 (0.83)
	Hx of surgery	-4.7 (0.59)
	Aortic root dim. z-sc.	-1.4 (0.61)
	Asc. aorta dim. z-sc.	0.1 (0.96)
	Adjusted R^2 (q)	-0.48 (0.93)
**AFV Pat.**	Diastolic dysfunction	-0.96 (0.95)
	Hx of surgery	-23.7 (0.5)
	Aortic root dim. z-sc.	-0.01 (0.0.99)
	Asc. aorta dim. z-sc.	6.3 (0.62)
	Adjusted R^2 (q)	-1.5 (0.88)
**Groups**	**Independent variables**	**Outcome variable: Taurine** **β/r^2 (q)**
**A Pat.**	Diastolic dysfunction	-14.6 (0.004)
	Hx of surgery	-34.4 (0.0002)
	Aortic root diam. z-sc.	-8.6 (0.0017)
	Asc. aorta dim. z-sc.	5.33 (0.0064)
	Adjusted R^2 (q)	0.83 (0.002)
**AF Pat.**	Diastolic dysfunction	-14.1 (0.005)
	Hx of surgery	-36.3 (0.0003)
	Aortic root dim. z-sc.	-8.1 (0.002)
	Asc. aorta dim. z-sc.	5.7 (0.005)
	Adjusted R^2 (q)	0.96 (0.002)
**AFV Pat.**	Diastolic dysfunction	-14.7 (0.15)
	Hx of surgery	-32.4 (0.13)
	Aortic root dim. z-sc.	-7.6 (0.12)
	Asc. aorta dim. z-sc.	5.7 (0.27)
	Adjusted R^2 (q)	0.9 (0.18)
**Groups**	**Independent variables**	**Outcome variable: PCaeC42:3** **β/r^2 (q)**
**A Pat.**	Diastolic dysfunction	-0.8 (0.28)
	Hx of surgery	-1.5 (0.17)
	Aortic root dim. z-sc.	-0.53 (0.15)
	Asc. aorta dim. z-sc.	0.45 (0.16)
	Adjusted R^2 (q)	0.03 (0.43)
**AF Pat.**	Diastolic dysfunction	-0.7 (0.29)
	Hx of surgery	-1.9 (0.08)
	Aortic root dim. z-sc.	-0.55 (0.11)
	Asc. aorta dim. z-sc.	0.54 (0.08)
	Adjusted R^2 (q)	0.18 (0.3)
**AFV Pat.**	Diastolic dysfunction	-14.2 (0.24)
	Hx of surgery	-1.7 (0.9)
	Aortic root dim. z-sc.	-3.4 (0.37)
	Asc. aorta dim. z-sc.	4.7 (0.45)
	Adjusted R^2 (q)	0.77 (0.32)
**Groups**	**Independent variables**	**Outcome variable: NT-proBNP** **β/r^2 (q)**
**A Pat.**	Diastolic dysfunction	14.6 (0.78)
	Hx of surgery	-4.2 (0.95)
	Aortic root dim. z-sc.	-3.7 (0.87)
	Asc. aorta dim. z-sc.	35.3 (0.11)
	Adjusted R^2 (q)	0.2 (0.26)

**AF Pat.**	Diastolic dysfunction<br>Hx of surgery<br>Aortic root dim. z-sc.<br>Asc. aorta dim. z-sc.	26 (0.4)<br>-48 (0.3)<br>-5.6 (0.7)<br>45.1 (0.01)
	Adjusted R^2 (q)	0.7 (0.02)
**AFV Pat.**	Diastolic dysfunction	-18.2 (0.38)
	Hx of surgery<br>Aortic root dim. z-sc.<br>Asc. aorta dim. z-sc.	6.7 (0.81)<br>-1.2 (0.85)<br>13.7 (0.36)
	Adjusted R^2 (q)	0.78 (0.31)
